# The impact of rhubarb (*Rheum Ribes* L.) juice-based marinade on the quality characteristics and microbial safety of chicken breast fillets during refrigerated storage

**DOI:** 10.1016/j.psj.2024.104719

**Published:** 2024-12-21

**Authors:** Pınar Karatepe, Gökhan Kürşad İncili, Ali Tekin, Mehmet Çalıcıoğlu, Müzeyyen Akgöl, Ali Adnan Hayaloğlu

**Affiliations:** a Department of Food Hygiene and Technology, Faculty of Veterinary Medicine, Fırat University, Elazığ, Türkiye; b Food Processing Department, Keban Vocational School, Fırat University, Elazığ, Türkiye; c Department of Food Engineering, Engineering Faculty, Inonu University, Malatya, Türkiye

**Keywords:** Rhubarb juice, Bioactive compounds, Chicken breast fillets, Cold storage, Foodborne pathogens

## Abstract

Acidic marinades are commonly used to improve the quality meat products. However, no study has been performed to determine the effects of rhubarb juice as a marinating liquid on the quality parameters of chicken breast fillets. The aim of the present study was to identify the bioactive compounds (organic acids, polyphenols, and volatiles) in the juice of rhubarb and to determine the effect of rhubarb juice as a marinade on the microbiological (total viable count, psychrotrophs, lactic acid bacteria, sulfate-reducing anaerobes, and yeast-molds) and physico-chemical properties (drip loss, cooking loss, water holding capacity, pH, color, malondialdehyde, total volatile base nitrogen, and texture profiles), sensory attributes, and microbial safety (*Escherichia coli* O157:H7, *Salmonella* Typhimurium, and *Listeria monocytogenes*) of chicken breast fillets during a 15-day refrigerated storage. The experiment included five groups: a control (no treatment), and marinated groups treated with 50 % and 100 % rhubarb juice for 6 and 24 h. The application of a rhubarb juice-based marinade (100 % for 24 h) resulted in a significant decrease in the number of *E. coli* O157:H7, *S.* Typhimurium and *L. monocytogenes* by 1.67 to 2.60 log_10_ cfu/g compared to the control group (*P* < 0.05). In addition, this marinade significantly reduced the growth of psychrotrophs, lactic acid bacteria and the total number of viable bacteria compared to the control group during storage (*P* < 0.05). The rhubarb juice-based marinade significantly delayed the increase in spoilage microorganisms and oxidation parameters compared to the control fillets (*P* < 0.05). No differences were found between the control and treatment groups in terms of sensory evaluation (*P* > 0.05). In conclusion, the results show that the juice of rhubarb juice contains a variety of organic acids, polyphenolic compounds and volatiles that contribute to antioxidant capacity and antimicrobial activity. In addition, the use of rhubarb juice as a marinating liquid delayed the oxidation of proteins and lipids, the growth of spoilage microorganisms and improved microbial safety by inhibiting foodborne pathogens in the chicken breast fillets.

## Introduction

The demand for marinated meat and meat products is increasing annually, leading to a corresponding expansion of the market for marinated products (Wei et al., 2022). Although the main aim of marinating is to improve sensory and textural properties, it can also be used to enhance the microbiological quality and safety of muscle foods. Marinating by immersion is an extensively used and easily accessible method that requires no special equipment and can be easily applied by consumers in their own kitchens. In this method, the meat part is immersed in a marinade for a certain period of time to achieve the desired aroma, taste, and tenderness. However, there are a number of variables to consider when trying to achieve the desired result, such as the composition of the marinade, the duration of the marinating process etc. (Şengün et al., 2021). The most commonly used components of marinades made from natural ingredients include salt, spices, oil, plant extracts, vegetable and fruit juices, wine, vinegar, essential oils, and dairy products, such as kefir and yogurt (Latoch et al., 2023; Osaili et al., 2024a, Osaili et al., 2024b). Therefore, studies on marinades with natural ingredients have gained attention in recent years (Budiarto et al., 2024). Thus, there is a need to research the effectiveness of marinades made from natural products.

The high susceptibility of chicken meat to microbiological spoilage is attributed to its ability to provide an optimal environment for microbial growth (Necidova et al., 2024). The presence or growth of microorganisms in chicken meat leads to significant economic losses and also contributes to a considerable proportion of foodborne diseases (Ekonomou et al., 2023; Moller et al., 2023). The European Food Safety Authority (EFSA, 2022) has published a report which states that poultry meat and meat products are a significant source of microbial foodborne diseases. *Campylobacter* spp., *Salmonella* spp., including *Salmonella enterica* subsp. *enterica* serovar Typhimurium, Shiga-toxigenic *Escherichia coli,* and *Listeria monocytogenes* are among the most common pathogens of microbial foodborne diseases originating from poultry products. The current state of microbiological problems in chicken meat has led to the conclusion that improving the microbiological quality and safety of chicken meat remains an attractive area of research.

The use of acidic marinades and marinating liquids derived from various plants has increased considerably in recent years. The effectiveness of various juices derived from apple and lemon (Augustyńska-Prejsnar et al., 2023), sour cherry and plum (Nour, 2022), pomegranate (Lytou et al., 2019), pineapple (Kadıoğlu et al., 2019), and lime (Lawrence and Lawrence, 2021) in marinating different types of meat has been investigated. The results showed that the use of acidic juices can enhance the textural, sensory, and microbiological quality of meat parts. However, to the best of our knowledge, no study has been performed to detect the effect of rhubarb juice as a marinating liquid on the quality parameters of chicken breast fillets.

The plant *Rheum ribes* L., commonly known as rhubarb, is the only member of the genus Rheum that grows in Turkey (Keser et al., 2020). A number of studies have demonstrated the presence of bioactive compounds and bioactivities in this plant, including organic acids, hydrocarbons and fatty acids. In addition, a variety of bioactive properties have been documented, including antioxidant and antimicrobial effects (Keser et al, 2020; Kalkan et al, 2020; Ceylan et al, 2019; İncili et al, 2021). In our previous research, a marinade based on rhubarb pulp enriched with eugenol and tymol was used and a considerable decrease in the number of some foodborne pathogens in chicken meat was observed (Karatepe et al., 2024). Nevertheless, further research on the potential of this plant is needed. Furthermore, no attempt has yet been made to use the juice of *Rheum ribes* L. as a marinade for chicken meat and meat products. The aim of the present study was to investigate the potential of the juice of this plant as a marinating liquid on the quality and safety characteristics of chicken breast fillets.

## Materials and methods

### Materials

Chicken breasts (supplied from a local slaughterhouse in Elazig province of Turkey) without skin in the initial stage of their shelf life were used for the study. The chicken breasts were transferred to the laboratory under refrigerated conditions within 2 h, and the experimental inoculation and marination treatments were performed on the same day. Samples of recently harvested rhubarb (*Rheum ribes* L.) were obtained from different local markets in Elazığ province of Turkey. The rhubarb was then washed, peeled and pressed into fresh rhubarb juice using a kitchen juicer.

### Methods

#### Characterization of the rhubarb juice

The organic acid composition of the rhubarb juice was quantified by a reversed-phase high performance liquid chromatography (**RP-HPLC**). For this purpose, 5 mL of freshly extracted rhubarb juice was centrifuged at 3000 × *g* for 10 min. Subsequently, the supernatant was passed through a 0.45 μm syringe filter. A 20 µL aliquot was injected into HPLC column, and the same chromatographic conditions according to our previously published research was used to determine the organic acids (İncili et al., 2023a). The results were expressed in milligrams per liter (mg/L). The polyphenols present in the rhubarb juice were identified using an HPLC system. A 250 µL of freshly extracted rhubarb juice was mixed with 10 mL of a methanol-water solution (85:15) and then subjected to ultrasonication for 10 min. Subsequently, the solution was mixed for 60 min under nitrogen gas. Afterwards, a centrifugation (7500 rpm for 10 min) was applied to the mixture, and then supernatant was filtered through a 0.45 μm pore size membrane filter. A 20 μL of aliquot was transferred to the ODS-3 column (C18, 250 × 0.5 mm × 0.5 μm), and the flow rate was set to 1 mL/min. Mobile phase A (HPLC grade water and 0.1 % H3PO4). Mobile phase B (HPLC grade water and 0.1 % acetonitrile) was adjusted as follows: from 0 to 25 % mobile phase B for 15 min, from 25 % to 75 % mobile phase B for 20 min, from 75 % to 100 % mobile phase B for 25 min. The isocratic flow was then reached at the 55th minute. The column was then conditioned by washing with methanol for 7 min. The column was eluted at 280, 320 and 360 nm, and the amounts of each compound are given in mg/L (İncili et al., 2023b). The Folin-Ciocalteu method was employed to determine the total phenolic content of the rhubarb juice in line with the methodology described in the study by İncili et al. (2022). In brief, a 0.8 μL sample of rhubarb juice (50 ppm) was mixed with the equal volume of Folin-Ciocalteu's reagent. Afterwards, 0.8 μL of Na₂CO₃ (20 %) was added to the mixture and kept at room temperature for 2 h. The total phenolic content was then measured at 760 nm (Shimadzu UV-1800, Kyoto, Japan). The results were expressed as Gallic Acid Equivalent per liter (GAE/L). The in vitro radical scavening activity of the rhubarb juice was determined by 2,2-diphenyl-1-picrylhydrazyl (**DPPH**) and 2,2-azino-bis-3-ethylbenzothiazoline-6-sulfonic acid (**ABTS**) radical methods outlined in our study (İncili et al., 2023b) and the results were expressed as mg Trolox Equivalent of Antioxidant Capacity (**TEAC**)/L. The volatiles present in the juice of rhubarb and chicken breast fillets (after 1st, 6th, and 15th days of refrigerated storage) were determined via solid-phase microextraction gas chromatography-mass spectrometry (**SPME/GC-MS**) method as described by İncili et al. (2021). Results were expressed as individual peak area of each compound compared to total peak area.

#### Preparation of chicken breast fillets, bacterial inoculum cocktail and experimental inoculation, and treatment groups

The skinned breast meat was cut with sterilized knives into small portions with a length of 10 cm, a width of 5 cm, and a height of 1 cm and an approximate weight of 100 ± 5 g. All breast fillets were then used in all experiments. All chicken breast fillets were then inoculated with foodborne pathogenic bacteria according to the methodology described below. The samples were inoculated with the strains *L. monocytogenes* (N 7144, ATCC 13932 and ATCC 7644), *Escherichia coli* O157:H7 (ATCC 43894, 35150 and 43895) and *S.* Typhimurium (ATCC 14028, NCTC 12416 and 74). Each bacterial strain was cultured in Tryptic Soy Broth at 37 °C for 24 h. Pellets and supernatant were then separated by centrifugation at 4200 × *g* for 10 min. The pellet was then resuspended in a solution of 0.1 % peptone water (2 mL). The absorbance of the resuspended pellet was adjusted to 0.1 at 600 nm, giving a value of approximately 8.0 log_10_ cfu/mL. Subsequently, 1 mL of each adjusted concentration of the tubes was collected in a single tube, which was then diluted tenfold, resulting in a concentration of 10^7^ log_10_ cfu/mL. This diluted tube was used for the experimental inoculation of the chicken breast fillets. For this purpose, 1 mL of the tenfold diluted inoculum cocktail (10^7^ log_10_ cfu/mL) was applied to the entire surface of the breast fillets (100 ± 5 g). The fillets were allowed to colonise the bacteria for 30 min at 4 ± 1 °C. The experimentally inoculated breast fillets were divided into five different groups. A control group was formed, which contained the samples that had not been subjected to any treatment. The treatment groups were exposed to two different concentrations of rhubarb juice (50 % and 100 %) for two different marinating times (6 and 24 h at 4 °C). An immersion marinade with a fillet to marinade ratio of 1:2 was used in the study (Karatepe et al., 2024). After the marinating time, all breast fillets were individually vacuum-packed and stored at a temperature of +4 °C for 15 days. A series of microbiological and physico-chemical analyses were carried out on days 1, 3, 6, 9, 12 and 15 during the entire storage period.

#### Microbiological analyses

A 25 g chicken meat sample was weighed and homogenized with 225 mL of 0.1 % sterile peptone water. Tenfold dilutions were prepared with 0.1 % peptone water. Total viable count (**TVC**) and psychrotrophic bacteria were counted using plate count agar (**PCA**, Biokar, France) according to the methodology described by the United States Department of Agriculture/Food Safety and Inspection Service (USDA/FSIS, 2015). The number of lactic acid bacteria (**LAB**), sulfate-reducing anaerobic bacteria, yeasts and molds was determined according to the methods described in the International Organization for Standardization (**ISO**) standards ISO 15214 (1998), ISO 15213 (2003) and ISO 21527 (2008), respectively. In addition, the number of *S.* Typhimurium, *L. monocytogenes* and *E. coli* O157:H7 was determined using the Xylose-Lysine-Tergitol-4 (**XLT**-4), Oxford and Cefixime-Tellurite-Sorbitol-MacConkey (**CT-SMAC**) agars, respectively. The inoculated plates were stored at 37 ± 2 °C for 24 h. After the incubation period, the number of colonies was recorded and converted to log_10_ cfu/g.

#### Determination of the rates of drip loss, cooking loss, and water holding capacity

The rates of drip loss (**DL**), cooking loss (**CL**) and water holding capacity (**WHC**) were determined using the methods described in the previously published studies (Karatepe et al., 2023; Kuttappan et al., 2016). For the determination of DL, 5 g of breast fillet was placed on filter paper in polyethylene trays and the trays were covered with stretch films. The sample was then incubated at 4 ± 1 °C for 3 days. The excess liquid on the surface of the breast fillets was then removed with paper towels. The DL was determined by calculating the weight changes between the initial and final weights. In the CL analysis, 10 g of chicken breast fillet was placed in a bag and cooked at 85 ± 1 °C for 10 min. After cooking, excess liquid on the surface of the breast fillets was removed with paper towels and then the weight of the sample was determined. The CL was determined by calculating the weight changes between the initial and final weights of the samples. For WHC analysis, 2 g of breast fillet was placed between filter paper and positioned between 10 × 10 cm glass plates. A weight of 10 kg was applied to the plate for 5 min. The changes in weight were then measured.

#### Determination of pH and *L, a* and *b*

For the pH analysis, 5±0.5 g of the breast fillet sample was homogenized with 45 mL of distilled water and the measurement was performed at 25 °C. The pH value of the fresh rhubarb juice was determined at 25 °C. Hunter's parameters (*L, a* and *b*) were used to determine the color characteristics of the breast fillets using a digital colorimeter (CR-5, Konica Minolta, Osaka, Japan) at a standard viewing angle of 10° with a illuminator (D-65). The measurements were taken on the entire surface of the breast fillets and at least 5 different measurements were taken from each sample.

#### Determination of malondialdehyde (MDA) and total volatile base‑nitrogen amounts (TVB-N)

The MDA content was determined by thiobarbituric acid (**TBA**) test according to the method of Wang et al. (2022). The determination of MDA was conducted by mixing 10 g of breast fillet with 25 mL of a 20 % trichloroacetic acid (**TCA**) solution (w/v). Afterwards, the mixture was centrifuged at 4200 × *g* for 10 min. Following centrifugation, the supernatant was filtered by using a filter paper, and then the equal volumes of the filtrate and 0.02 mol/L TBA reagent were combined. The mixture was then incubated at 85 °C for 30 min. Following incubation, the mixture was cooled to 25 °C, and the absorbances were measured at 532 nm. The results expressed as mg/malonaldehyde (MDA)/kg. Total volatile basic nitrogen analysis was performed by the method outlined in our previous study and the results were expressed as mg/100 g (İncili et al., 2022).

#### Determination of textural profiles of the chicken breast fillets

The hardness, springiness, cohesiveness, gumminess, chewiness, and resilience values of the raw chicken breast fillets were determined using a texture analyzer (TA-XT plus, UK) in accordance with the method described in a study conducted by Akdeniz İncili et al. (2023).

#### Sensory evaluation of the chicken breast fillets

The sensory evaluation was carried out on non-inoculated breast fillets (control and marinated). A total of ten panelists evaluated the cooked breast fillets. The breast fillets were cooked in a kitchen-type oven individually at 180 ± 5 °C for 25 min. Following this, the breast fillets were served to the panelists with randomly assigned numbers that gave no indication of the groups used in the experimental design. The cooked breast fillets were presented to the panel members of the Department of Food Hygiene and Technology of the Faculty of Veterinary Medicine of Fırat University, Turkey (academic staff and PhD students). The panellists had experience in marinating and had participated in the sensory evaluation of marinated meat and meat products. The sensory panel was conducted in daylight (at 14:00) and participants were served room temperature water and unsalted crackers. A five-minute break was taken between evaluations of the samples to reduce the margin of error in sensory interpretation. Sensory evaluations were made using a numerical scale from 1 (extremely poor) to 9 (extremely good) for color, odor, appearance, texture, taste and overall acceptability (Şengün et al., 2021).

### Statistical analyses

Three independent replicates were used for all analyses in the experimental design, and data were given as mean and standard error (**SE**). SPSS package program version 21.0 was used for statistical analyses, and the significance threshold was set at 0.05. The statistical technique of analysis of variance (two-way **ANOVA**) with post hoc Tukey's test was employed for the purpose of conducting multiple comparisons between groups and sampling days.

## Results and discussion

### The composition of the rhubarb juice

A total of four different organic acids were identified in fresh rhubarb juice. The predominant organic acid was citric acid. This was followed by malic and lactic acids, as shown in Table 1. These acids are the main contributors to the low pH (3.59±0.03) and antimicrobial activity of the juice. In agreement with our results, Stoleru et al. (2019) also identified the organic acids as the main constituent of another rhubarb variety (*Rheum rhabarbarum* L.). The principal compounds in rhubarb samples are organic acids, although the composition of these acids may vary between different rhubarb varieties. These differences can also be influenced by a number of factors, including the type of rhubarb, the growing conditions, the geographical region where the rhubarb is grown and the different parts of the plant, such as the stem and root (Will and Dietrich, 2013; Kharchenko et al., 2023; Stoleru et al., 2019).

**Table 1:** Organic acids present in the *Rheum ribes* L. (rhubarb) juice (mean ± SE).

Organic acid	Amount (mg/L)
Citric acid	19163.27 ± 1231.59
Malic acid	6953.50 ± 459.89
Lactic acid	3358.62 ± 432.92
Acetic acid	219.29 ± 177.59

In the current study, 78 different volatile organic compounds (**VOCs**) were determined in the fresh juice of *Rheum ribes* L. (rhubarb), as shown in Table 2. The main groups of VOCs were alcohols, terpenes and esters. 3-Hexen-1-ol and 1-hexanol were the predominant alcohols with the highest peak areas among the volatiles. The alcohols are known to exhibit antimicrobial activity against a variety of microorganisms. In this context, one study reported that exposure to 3-hexen-1-ol and 1-hexanol inhibited the growth of *Fusarium* strains (Cruz et al., 2012). In addition, terpenes were identified as the second most abundant volatile group in the fresh rhubarb juice. Among the terpenes, linalool was identified as the most abundant component. This is a well-characterised cyclic monoterpene with proven anticarcinogenic and antimicrobial properties (Masyita et al., 2022).

**Table 2:** Volatile compounds identified in the *Rheum ribes* L. (rhubarb) juice (mean peak area ± SE).

Volatiles	Peak Area
***Aldehydes***	
Acetaldehyde	4.19 ± 0.21
Hexanal	4.11 ± 0.47
2-Hexen-1-al	3.84 ± 0.11
Benzaldehyde	1.15 ± 0.31
Octanal	0.60 ± 0.14
Nonanal	4.08 ± 1.07
***Total***	***17.97***
***Alcohols***	
Ethanol	16.03 ± 0.75
1-Penten-3-ol	1.90 ± 0.23
3-Methyl-1-butanol	1.27 ± 0.25
2-Methyl-1-butanol	0.27 ± 0.04
1-Pentanol	0.44 ± 0.04
2-Penten-1-ol	3.68 ± 0.34
3-Hexen-1-ol	352.40 ± 30.62
1-Hexanol	37.10 ± 2.47
3,5-Hexadien-1-ol	0.29 ± 0.03
1-Heptanol	0.59 ± 0.04
1-Octen-3-ol	2.64 ± 0.52
2-Ethyl-hexanol	6.66 ± 0.95
Non-2-enol	1.57 ± 0.64
1-Octanol	0.78 ± 0.04
4-Nonanol	0.45 ± 0.05
2,6-Dimethyl-2,6-octadiene-1,8-diol	0.50 ± 0.03
Methol	0.58 ± 0.04
Nonylol	0.42 ± 0.01
1-Decanol	0.66 ± 0.07
5,7-Undecadienol	0.29 ± 0.06
1-Dodecanol	0.40 ± 0.05
***Total***	***428.91***
***Terpenes***	
Oxime-methoxy-phenyl	2.03 ± 0.32
2-β-Pinene	2.45 ± 0.83
β-Myrcene	4.69 ± 1.03
l-Limonene	26.37 ± 8.97
1,8-Cineole	32.96 ± 9.96
β-Ocimene	0.79 ± 0.14
γ-Terpinen	0.37 ± 0.11
Dihydromyrcenol	0.64 ± 0.08
*p*-Cymenene	0.68 ± 0.18
Linalool	39.37 ± 6.88
*p*-Menthan-3-one	1.15 ± 0.26
*p*-Menthone	0.50 ± 0.07
4-Terpineol	1.19 ± 0.02
*p*-Menth-1-en-8-ol	11.38 ± 0.92
*p*-Ment-6-en-2-one	1.97 ± 0.31
Dehydrogeosmin	0.68 ± 0.10
Thujopsene	0.38 ± 0.01
***Total***	***127.6***
***Ketones***	
2-Pentanone	0.40 ± 0.07
3-Pentanone	1.30 ± 0.13
3-Heptanone	1.17 ± 0.07
4-Octanone	0.49 ± 0.04
3-Octanone	3.98 ± 1.23
***Total***	***7.33***
***Esters***	
Ethyl Acetate	19.55 ± 6.61
Methyl-2-methyl-butanoate	0.75 ± 0.06
Diisooctyl diphosphorate	0.59 ± 0.11
3-Tridecyl methoxyacetate	10.47 ± 1.94
Methyl 13-docosenoate	0.82 ± 0.12
Methyl octanoate	1.30 ± 0.28
4-Tetradecyl chloroacetate	0.58 ± 0.08
10-Undecenyl pentanoate	0.65 ± 0.06
***Total***	***34.72***
***Hydrocarbons***	
2,4-Dimethyl-pentane	1.73 ± 0.21
3,3-Dimethyl-oxetane	0.62 ± 0.07
Styrene	0.47 ± 0.06
o-Xylene	0.46 ± 0.10
1,1′-Oxybis-decane	0.31 ± 0.02
1-Dodecene	0.35 ± 0.01
3-Dodecene	0.61 ± 0.30
3,4-Decadiene	0.65 ± 0.04
Tetradecane	1.41 ± 0.07
***Total***	***6.61***
***Miscellaneous compounds***	
Methanethiol	0.90 ± 0.06
Dimethyl sulfide	1.78 ± 0.21
Dichloro methane	4.59 ± 0.59
Trimethyl-silanol	2.54 ± 0.41
2-Ethyl-furan	0.45 ± 0.04
Dimethyl-silanediol	1.30 ± 0.27
Toluene	1.90 ± 0.62
Pentanoic acid	1.20 ± 0.45
Methyl salicylate	2.77 ± 0.27
Diethyl phthalate	0.57 ± 0.12
2,4-Bis(1,1-dimethylethyl)-phenol	1.18 ± 0.35
***Total***	***19.18***

A total of 23 phenolics and flavonoids were identified in the juice of *Rheum ribes* L., with rutin being the most abundant compound (Table 3). Plant-derived polyphenols have been shown to possess a variety of bioactive properties, such as anti-inflammatory, antidiabetic, anti-obesity, anticarcinogenic, antimicrobial, antiallergic, and antioxidant effects (Luca et al., 2019; Mitra et al., 2022). The results of the present study showed that rhubarb juice is a rich source of phenolic acids, including vanillic, gallic, protocatechic and 4-dihydroxybenzoic acids, as well as flavonoids such as epicatechin, rutin and quercetin-3-glucoside. Furthermore, the high polyphenol content of rhubarb juice was confirmed by TPC analysis, which showed a concentration of 947.26±13.70 mg GAE/L. Polyphenolic compounds are known to contribute to antioxidant capacity, as evidenced by the strong free radical scavenging activity of rhubarb juice documented by DPPH (26150.60±1135.77 mg TEAC/L) and ABTS (10462.6±6.00 mg TEAC/L) analyses. A comparable result was documented by Kharchenko et al. (2023), who reported that different varieties of rhubarb (*Rheum rhabarbarum* L.) exhibited considerable free radical scavenging activity. In contrast, the antioxidant capacity of rhubarb juice in terms of DPPH and ABTS was significantly higher than that of juices used as marinating liquids in previous studies, including homemade fermented pickle juice (Akdeniz İncili et al., 2023) and hawthorn vinegar (Karatepe et al., 2023). The high antioxidant capacity of rhubarb juice has been shown to delay the oxidation of lipids and proteins in food.

**Table 3:** Individual phenols and flavonoid compunds found in the *Rheum ribes* L. (rhubarb) juice (mean ± SE).

Compound	Amount (mg/L)
Gallic acid	6.32 ± 0.13
Vanilic acid	34.50 ± 0.37
Protocatechuic acid	0.75 ± 0.01
Procyanidin B2	1.71 ± 0.20
Catechin	4.25 ± 0.08
4-Hydroxy benzoic acid	16.62 ± 0.35
Syringic acid	0.77 ± 0.03
Epicatechin	0.21 ± 0.01
Hesperidin	0.14 ± 0.01
Caftaric acid	2.94 ± 0.00
Chlorogenic acid	1.76 ± 0.00
2,5-Dihydroxy benzoic acid	1.68 ± 0.00
*t-*Caffeic acid	0.80 ± 0.02
*p*-Coumaric acid	1.88 ± 0.01
Sinapic acid	1.67 ± 0.03
Ferulic acid	1.61 ± 0.01
Resveratrol	0.21 ± 0.00
Rutin	127.33 ± 2.63
Quercetin-3-glucoside	2.85 ± 0.04
Kaempferol-3-glucoside	1.30 ± 0.47
Myricetin	1.32 ± 0.01
Quercetin	1.00 ± 0.00
Luteolin	5.19 ± 0.03

### Microbiological results of the rhubarb juice-based marinated chicken breast fillets

**Fig. 1:**
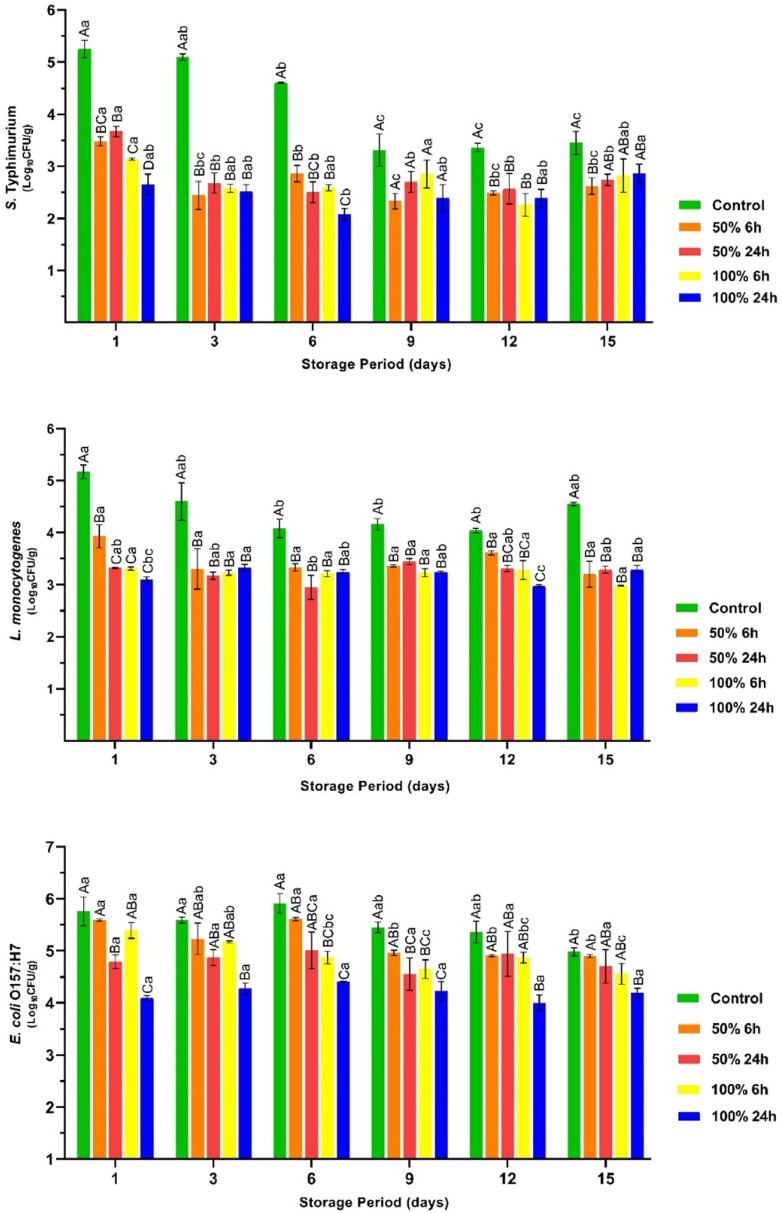
The count for *Salmonella* Typhimurium, *Listeria monocytogenes*, and *Escherichia coli* O157:H7 in the vacuum-packaged marinated with rhubarb juice and non-marinated chicken breast fillets during storage period at 4 °C (mean ± SE). ^A–C^: The mean values with different letters between the groups, ^a–c^: The mean values with different letters between the sampling days are significantly different (*P* < 0.05).

The current study shows that a marinade based on rhubarb juice causes a considerable reduction in the number of pathogenic bacteria (Fig. 1, *P* < 0.05). In addition, the inhibition rate increased with the concentration of rhubarb juice and the marinating time. On the first day, the highest inhibition rates were observed in chicken breast fillets marinated with 100 % rhubarb juice for 24 hours. The populations of *S.* Typhimurium, *E. coli* O157:H7 and *L. monocytogenes* were 2.60, 1.67 and 2.08 log_10_ cfu/g lower in this group than in the untreated fillets (*P* < 0.05). The observed antibacterial effect may be attributed to the presence of organic acids in the rhubarb juice (Table 1) and the low pH of the juice (3.59). In agreement with our results, other researchers have also reported a comparable or higher reduction in the number of *E. coli* O157:H7, *S.* Typhimurium, and *L. monocytogenes* in various meats treated with acidic marinades containing fermented pickle juice, hawthorn and fruit vinegar and koruk juice (Akdeniz İncili et al., 2023; Karatepe et al., 2023; Ozturk and Sengun, 2019; Sengun et al., 2021). Nevertheless, a variety of factors such as the pH of the marinating liquid, the type and amount of organic acids contained in the marinades, the marinating duration, the type of meat and the acid sensitivity of the target bacteria can have a remarkable effect on the inhibition rates of foodborne pathogens by marinating. The results of this study show that marinating with rhubarb juice has the potential to improve the microbiological safety of chicken breast fillets with regard to the inhibition of foodborne pathogens.

**Fig. 2:**
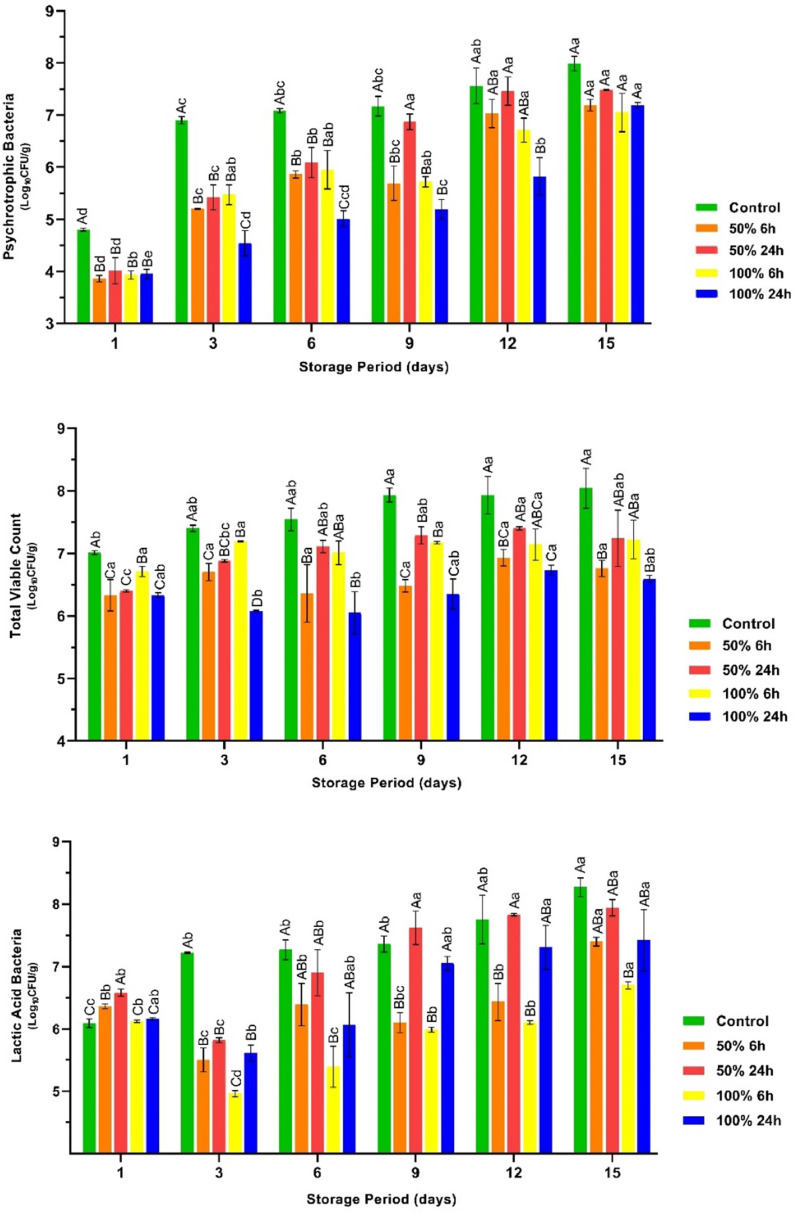
The count for psychrotrophic bacteria, total viable count, and lactic acid bacteria counts in the vacuum-packaged marinated with rhubarb juice and non-marinated chicken breast fillets during storage period at 4 °C (mean ± SE). ^A–D^: The mean values with different letters between the groups, ^a–d^: The mean values with different letters between the sampling days are significantly different (*P* < 0.05).

Conversely, the number of pathogenic bacteria was found to exhibit dynamic variability throughout storage. In particular, the number of *S.* Typhimurium and *L. monocytogenes* decreased in the control group during the storage period. It has been proven that microbial spoilage of meat and meat products is the result of dynamic interactions between different microbial groups (Saenz-Garcia et al., 2020). The rapid dominance of microbial groups during the spoilage process suppresses the proliferation of low-competitive bacteria, such as *S.* Typhimurium. Consistent with this information, a considerable increase in the number of psychrotrophs, TVC and LAB was observed in the control group during storage, as shown in Fig. 2 (*P* < 0.05). The exponential proliferation of these microorganisms could be the cause of the observed decrease in the number of *S.* Typhimurium and *L. monocytogenes* in the control group during storage. Although the control group had a significantly higher growth rate for psychrotrophs compared to the treatment groups, the rhubarb juice-based marinade resulted in a significant slowdown in the growth of these microorganisms. On the other hand, the present study showed that the TVC count in the control group already reached the upper limit (7.0 log_10_) set by the ICMSF on the first day of the study (ICMSF, 2002). However, it should be noted that the use of three pathogenic bacteria and three strains each for the experimental inoculation of the breast fillets at about 5.0 ± 0.5 log_10_ may have contributed to the high initial TVC counts for chicken breast fillets. The breast fillets marinated with 100 % concentration of rhubarb juice for 24 h remained below the upper TVC limit throughout the storage period.

**Fig. 3:**
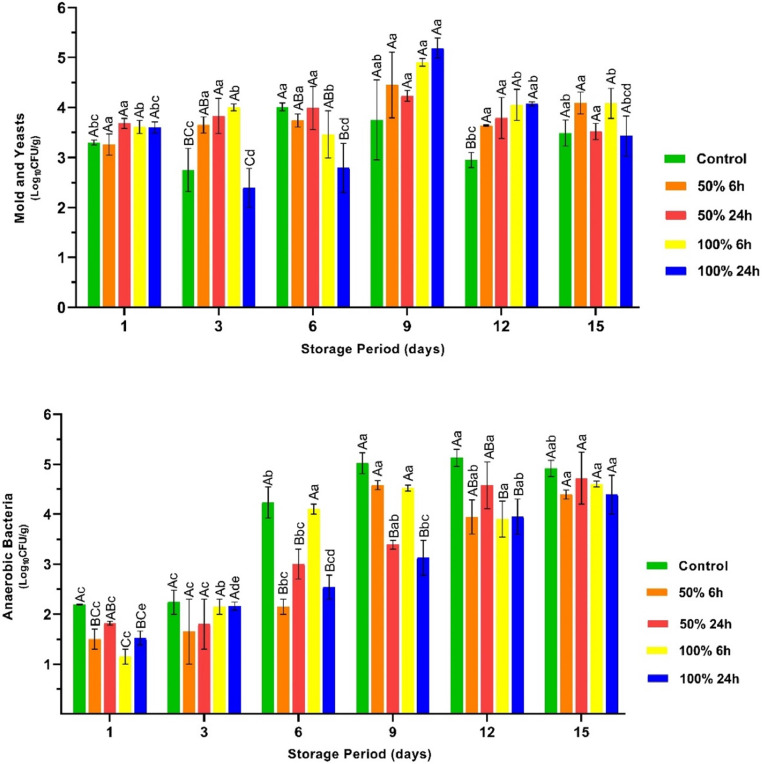
The count for mold and yeasts, and anaerobic bacteria counts in the vacuum-packaged marinated with rhubarb juice and non-marinated chicken breast fillets during storage period at 4 °C (mean ± SE). ^A–C^: The mean values with different letters between the groups, ^a–e^: The mean values with different letters between the sampling days are significantly different (*P* < 0.05).

It is well documented that LAB strains, including those belonging to the genera *Lactobacillaceae* and *Lactobacillus*, are one of the dominant microbial groups involved in the spoilage of vacuum-packed poultry meat (Juszczuk-Kubiak et al., 2021). In accordance with this information, the number of LAB in the untreated chicken breast fillets exhibited a remarkable increase during storage (*P* < 0.05). Nevertheless, a slower growth rate was observed in the marinated fillets, except for the breast fillets that were marinated with 50 % of rhubarb juice for 24 h. It is noteworthy that the breast fillets marinated with 50 % of the rhubarb juice for 24 h had similar or higher LAB values than those of the control group after the 3rd d of storage. Although the two-fold dilution of the rhubarb juice did not lead to a significant decrease in pH (3.63), the content of organic and phenolic acids involved in antimicrobial activity decreased by half. The two-fold decrease in the concentration of these compounds led to a considerable reduction in the antimicrobial efficacy of rhubarb juice against the LAB present in the original microflora of the breast fillets. In addition, a long marinating period (24 h) could have led to the colonization of acid-resistant bacteria in the microflora of the chicken breast fillets, resulting in insufficient inactivation. On the first day of the experimental design, the number of molds and yeasts in the chicken breast fillets marinated with 50 % (24 h) and 100 % (6 and 24 h) rhubarb juice was found to be slightly higher than in the other groups (Fig. 3; *P* > 0.05). It is well known that certain yeast and mold strains have a greater resistance to low pH than bacteria (Lund et al., 2020). In line with this information, the use of an acidic marinade with a low pH and a longer marinating time may have favored the initial growth of yeasts and molds. In addition, the rapid increase of psychrotrophs and LAB in the control group after the third day of storage is thought to be the reason that these bacteria dominated the microflora of the chicken breast fillets, thereby inhibiting the growth of yeasts and molds. In conjunction with this information, it was observed that the groups with higher numbers of psychrotrophic bacteria and LAB had lower numbers of molds and yeasts throughout the storage period (Fig. 2, Fig. 3).

The results of this study showed that the number of anaerobic bacteria in the control group was consistently higher than in the other groups throughout the storage period (*P* < 0.05). It is known that sulphate-reducing bacteria play an important role in the spoilage of vacuum-packed meat and meat products (Esteves et al., 2021). The interruption of oxygen contact with vacuum packaging allows anaerobic bacteria to become the dominant microorganisms. In light of this information, a significant increase in anaerobic bacteria was observed in all breast fillets after the third day in the present study. However, it was found that the increase was more pronounced in the control group than in the other groups (*P* ˂ 0.05).

### Effects of rhubarb juice-based marinade on pH, TBA and TVB-N of the chicken breast fillets

**Fig. 4:**
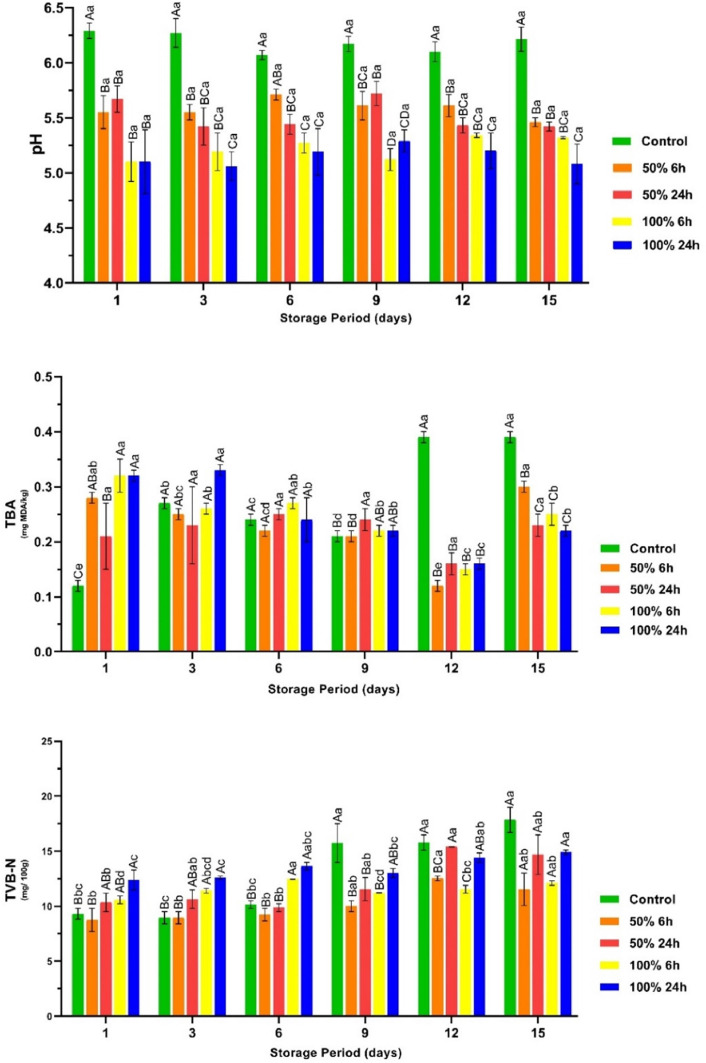
The pH, TBA and TVB-N values of the vacuum packaged marinated with rhubarb juice and non-marinated chicken breast fillets during storage period at 4 °C (mean ± SE). ^A–D^: The mean values with different letters between the groups, ^a–e^: The mean values with different letters between the sampling days are significantly different (*P* < 0.05).

The pH value is a decisive quality parameter for assessing the freshness of meat and meat products (Liu et al., 2021). The acidic marinade used in the current study significantly lowered the pH of the breast fillets, and the extent of this lowering depended on the concentration of rhubarb juice and marinating time, as shown in Fig. 4 (*P* < 0.05). It was observed that the pH values of the chicken breast fillets exhibited slight changes throughout the storage period (*P* > 0.05). The observed differences between the groups on the first day of the experimental design remained constant throughout the storage period. The observed stability across all groups can be attributed to the limitation of oxygen contact due to the use of vacuum packaging and the rapid increase in the number of groups such as LAB, which play a role in degradation, especially under anaerobic conditions, and become dominant in the microflora.

It is well established that TBA and TVB-N are used as indicators of oxidative spoilage. The present study showed that the application of a rhubarb juice-based marinade resulted in a considerable increase in TBA levels compared to the untreated samples on the initial day (Fig. 4, *P* < 0.05). However, it is postulated that this increase is a consequence of the denaturation of proteins by the use of an acidic marinade and the subsequent release of iron in the sarcoplasmic protein and myoglobin and not the result of oxidative degradation (de Avila Souza et al., 2022). The release of iron from the protein complex may have led to an increase in TBA and TVB-N levels in the breast fillets marinated with rhubarb juice on the first day of storage (*P* < 0.05). The MDA values showed a partial decrease in the breast fillets treated with rhubarb juice by day 12 (*P* < 0.05). In contrast, the control group exhibited a rapid increase, especially after the 9th day of storage (*P* < 0.05). As mentioned above, the antioxidant capacity of rhubarb juice could be responsible for the observed decrease in the rhubarb juice-treated groups. Conversely, despite the dramatic increase in growth observed in the control group, none of the samples exceeded the upper limit of 1.0 mg/kg for MDA levels (Chaijan et al., 2021). This can be attributed to the restriction of oxygen contact due to vacuum packaging. For TVB-N results, a rapid increase was observed in the control group during the storage period (*P* < 0.05), while the marinated chicken breast fillets showed comparatively little change, except for the breast fillets marinated with a 50 % concentration of rhubarb juice for 24 h. The slight changes observed in the marinated breast fillets can be attributed to the antioxidant capacity of the rhubarb juice, as shown by the data from the DPPH and ABTS analyses. A number of microbial groups that contribute to spoilage of chicken meat, such as psychrotrophs and LAB, were found to be more prevalent in this group than in the control group (Fig. 2). It is hypothesised that the proteolytic activities of these microorganisms are responsible for the observed increase in TVB-N levels during storage in these groups (García-Cano et al., 2019). Similar to the MDA results, none of the samples exceeded the upper TVB-N limits (25 mg/100 g) (Khazaei et al., 2024).

### Volatile profiles of the chicken breast fillets

**Fig. 5:**
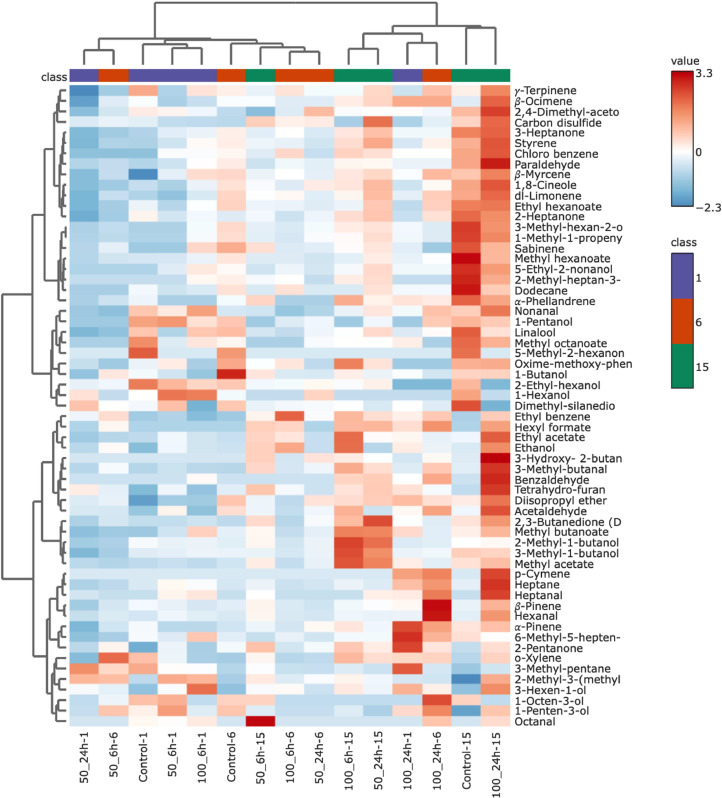
Volatile profiles of vacuum packed marinated with rhubarb juice and non-marinated chicken breast fillets during storage at 4 °C on days 1, 6 and 15 of the storage peroid.

Spoilage of fresh meat is a complex process involving a variety of factors, including microbiological, chemical, and enzymatic reactions. These reactions lead to changes in the physical, chemical, and sensory properties of meat and meat products. A variety of factors, such as the type and level of microorganisms present in the initial microbial load, storage conditions, handling procedures and the type of packaging, have been shown to have a significant impact on the spoilage process (Mansur et al., 2019). Alcohols, ketones, hydrocarbons, aldehydes and sulphur compounds have been shown to be the main contributors to the off-flavours observed in meat during the spoilage process (Santos et al., 2021). In accordance with this information, there was a significant increase in the content of alcohols (ethanol, 3-methyl-1-butanol and 1-hexanol), aldehydes (acetaldehyde and hexanal), esters (methyl acetate, ethyl acetate, methyl acetate, ethyl acetate, methyl butanoate and methyl hexanoate), hydrocarbons (chlorobenzene, styrene and dodecane) and ketones (3-heptanone, 2-heptanone and 3-methylhexan-2-one) in the control group, as shown in Fig. 5 and Supplementary Table 1. The formation and accumulation of these volatiles in chicken meat during spoilage is responsible for the development of undesirable odours. For instance, 3-methyl-1-butanol has been shown to lead to a whiskey-like odour, while 1-hexanol is associated with a faint metallic odour (Santos et al., 2021). Conversely, the marinated chicken breast fillets had higher levels of acetaldehyde and hexanal than the control group. This is due to the presence of these compounds in the rhubarb juice, as shown in Table 2. In addition, the levels of ethanol and 3-methyl-1-butanol in the non-marinated breast fillets increased dramatically during the storage period. In the marinated groups, however, these compounds were already detected in high concentrations on the first day of storage. Like the aldehydes already mentioned, these alcohols are also among the compounds identified in the volatile profile of the rhubarb juice. Certain esters, including methyl acetate, ethyl acetate, methyl butanoate, and methyl hexanoate, were found to increase significantly in the non-marinated fillets over the 15-day storage period. However, the initial concentration of ethyl acetate was higher in the marinated breast fillets than in the control group. This increased value can be attributed to the initial increased content in the marinated breast fillets, which is the predominant ester compound in the volatile profile of the rhubarb juice. Certain ketones, such as 3-methyl-hexan-2-one, 2-heptanone and 2-methyl-heptan-3-one, showed a notable increase in the control group during the storage period. In addition, the concentration of 3-heptanone was found to increase approximately fivefold in the control group between the first and last day of storage. The accumulation of these volatiles could be the main reason for the off-odour, which is why the breast fillets of the control group were no longer sensory evaluated after the 9th d of storage (see Table 5).

### Effects of the rhubarb juice-based marinade on pyschicochemical characteristics of the chicken breast fillets

**Fig. 6:**
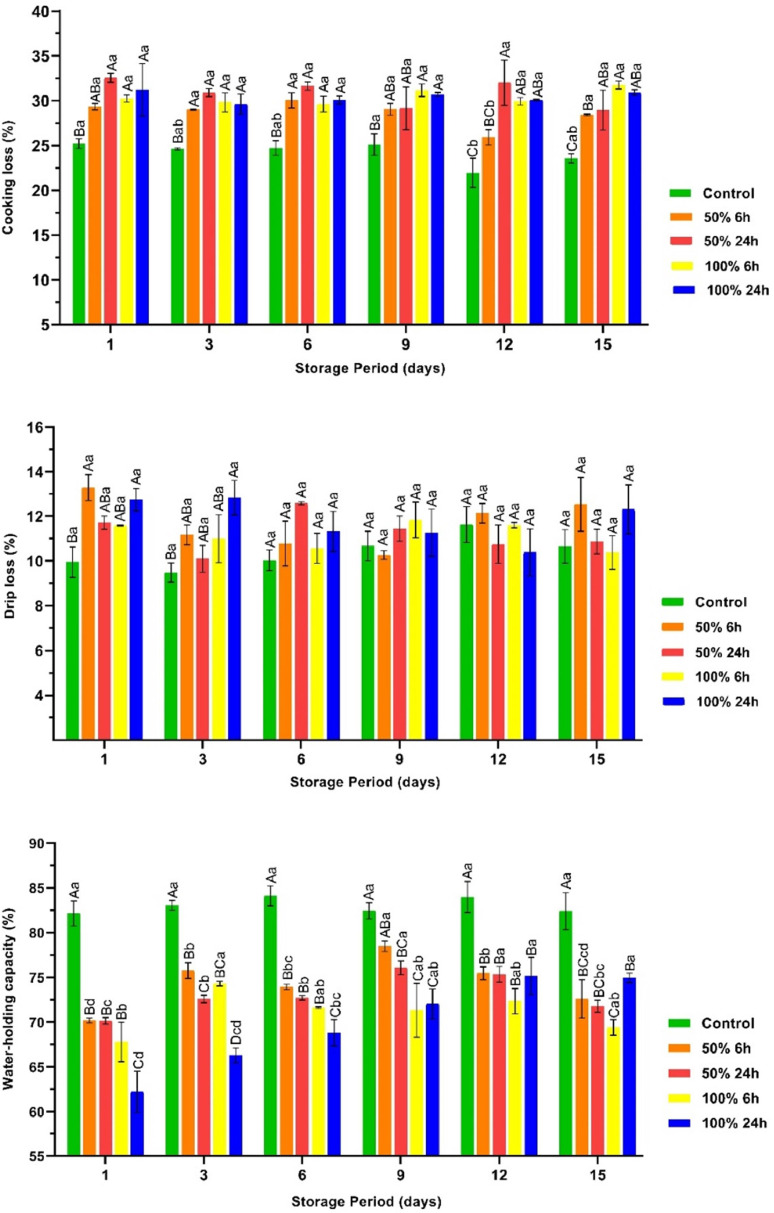
The cooking loss, drip loss, and water-holding capacity values of the vacuum packaged marinated with rhubarb juice and non-marinated chicken breast fillets during storage period at 4 °C (mean ± SE). ^A–D^: The mean values with different letters between the groups, ^a–d^: The mean values with different letters between the sampling days are significantly different (*P* < 0.05).

The CL, DL and WHC results of the chicken breast fillets are shown in Fig. 6. The results indicated that marinating causes significant changes in all three parameters (*P* < 0.05). It was found that the marinated chicken breast fillets showed an increase in both DL and CL in contrast to the non-marinated breast fillets. In contrast, the WHC value showed a decrease compared to the non-marinated samples (*P* < 0.05). These parameters are directly correlated with the pH value of the meat. It is well known that proteins lose water as they approach the isoelectric point (Akdeniz İncili et al., 2023). In this study, it was observed that the pH of the marinated breast fillets was closer to the isoelectric point (5.3) than that of the control group (Fig. 4). The observed increase in DL and CL as well as the decrease in WHC can be attributed to the proximity of the isoelectric point. The results of the current research study are consistent with previous studies. For instance, marination with hawthorn vinegar and homemade fermented pickle juice resulted in a significant decrease in DL and CL values of the beef steaks (Karatepe et al., 2023; Akdeniz İncili et al., 2023). Although no remarkable change in WHC value was observed in the control group during the storage period, some changes were observed in the marinated fillets (*P* < 0.05). These slight changes can be attributed to the insignificant changes in pH values observed in all breast fillets during storage, as shown in Fig. 4.

**Fig. 7:**
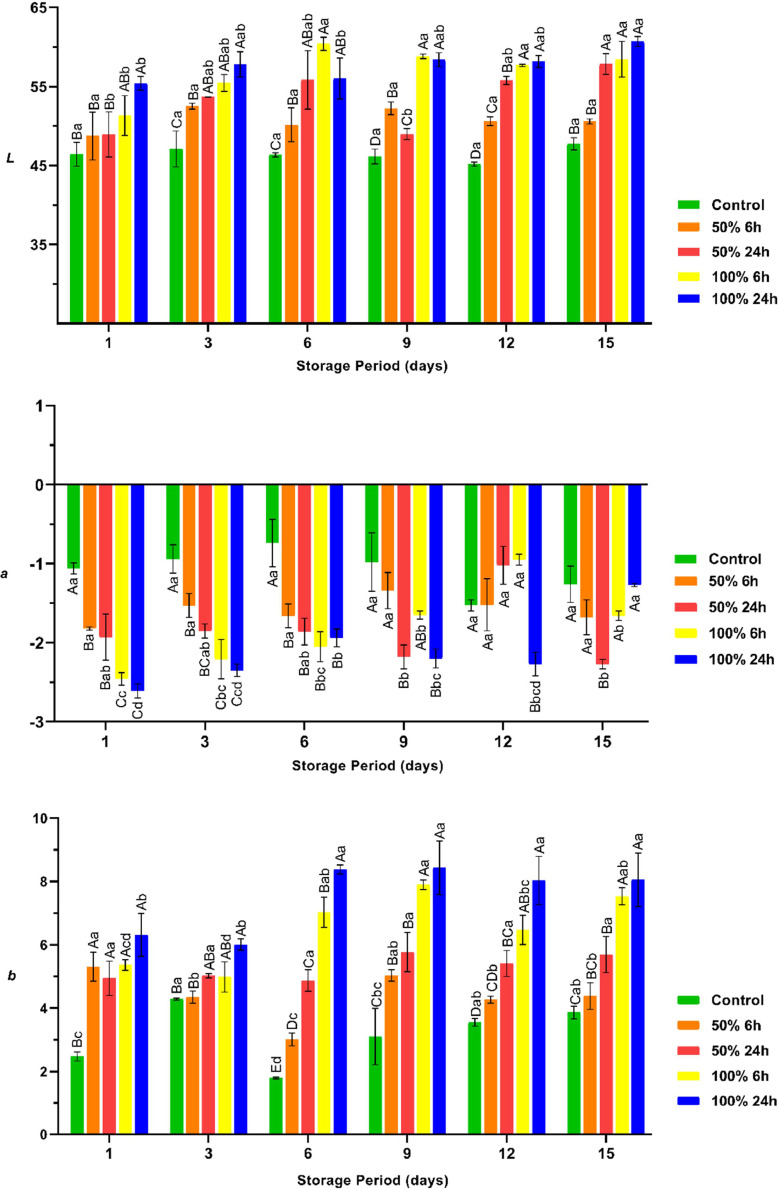
The *L, a*, and *b* values of the vacuum packaged marinated with rhubarb juice and non-marinated chicken breast fillets during storage period at 4 °C (mean ± SE). ^A–E^: The mean values with different letters between the groups, ^a–d^: The mean values with different letters between the sampling days are significantly different (*P* < 0.05).

The results of the present study show that marinating with rhubarb juice has a significant effect on the colour characteristics (*L, a,* and *b*) of raw chicken breast fillet, as shown in Fig. 7 (*P* < 0.05). The interaction between pH and meat colour is well documented, and there is evidence that meat with low pH and WHC has high *L* value (Jankowiak et al., 2021; Chen et al., 2018). In line with this information, the present study showed that the breast fillets with lower pH values had higher *L* and *b* values than those with higher pH values. The reason for the observation that the breast fillets with low pH and low WHC had high *L* values can be attributed to the fact that these samples release water as they approach the isoelectric point, resulting in a watery surface that appears brighter. In addition, the application of a rhubarb juice-based marinade resulted in a significant reduction in *a* values compared to the control group (*P* < 0.05). The changes in the *a* values can be attributed to the conversion of myglobin. It has been documented that acidification affects the conversion of myoglobin to metmyoglobin, which can lead to a change in the redness (*a* value) of the meat and meat products (de Avila Souza et al., 2022). On the other hand, it was found that the differences between the groups in terms of colour parameters remained constant throughout the storage period. As previously mentioned, the pH of the breast fillets remained stable throughout the storage period. These results provide an explanation for the stability of the observed differences in colour parameters between the groups throughout the storage period.

### Changes in the texture paramaters of the breast fillets

The texture parameters of the chicken breast fillets in the current study are shown in Table 4. It is known that there is a negative correlation between hardness and WHC values (Jankowiak et al., 2021). In line with this information, the chicken breast fillets with low WHC values showed higher hardness values, while those with high WHC values showed lower hardness values (Fig. 6 and Table 4). In addition, the WHC and hardness values in the control group remained constant throughout the storage period (*P* > 0.05). However, the WHC values of the breast fillets marinated with a 100 % concentration of rhubarb juice for 24 h increased during storage. In this group, a significant decrease in hardness value was observed on day 15 compared to day 1 (*P* < 0.05). As previously mentioned, approaching the isoelectric point of pH leads to a loss of water from the meat, which in turn leads to an increase in hardness. Conversely, a shift away from the isoelectric point leads to greater water absorption by the meat, which consequently increases tenderness (Akdeniz İncili et al., 2023). In accordance with this information, the pH of the breast fillets marinated with 100 % concentration of rhubarb juice for 6 and 24 h showed the closest proximity to the isoelectric point, and these fillets had the highest hardness, gumminess, chewiness and resilience values compared to the others. On the other hand, the values for hardness, springiness, cohesiveness, gumminess, chewiness and resilience of the breast fillets decreased in all groups during storage (*P* < 0.05). The observed decreases in the groups are probably due to a remarkable increase in the number of microorganisms with proteolytic activity, including psychrotrophs, LAB, yeasts and molds (Fig. 2, Fig. 3). It can be hypothesized that the textural properties of the breast fillets may be influenced by the proteolysis induced by microorganisms and autolytic enzymes.

**Table 4:** Changes in the texural properties in the non-marinated and the chicken breast fillets marinated with rhubarb juice during storage at 4 °C (mean ± SE).

Groups						
Storage (Days)		Control	50 % 6h	50 % 24h	100 % 6h	100 % 24h
**Hardness (N)**	**0**	6465.81 ± 743.64^BCa^	5462.40 ± 62.86^Cb^	5687.20 ± 398.71^BCb^	6929.60 ± 429.55^Bc^	9856.69 ± 346.01^Aa^
	**3**	5101.41 ± 1065.89^BCa^	6024.90 ± 343.28^BCb^	6939.61 ± 401.55^ABab^	4754.45 ± 258.73^Cd^	8305.39 ± 889.92^Aab^
	**6**	5877.43 ± 777.24^Ba^	5241.08 ± 542.08^Bb^	5408.98 ± 761.30^Bb^	7365.64 ± 445.80^ABbc^	8360.17 ± 894.88^Aab^
	**9**	6280.66 ± 693.80^BCa^	6111.64 ± 178.11^Cb^	7895.91 ± 528.71^ABa^	8160.60 ± 723.45^Aabc^	8680.80 ± 509.71^Aab^
	**12**	5383.01 ± 1071.15^Ca^	8050.57 ± 590.44^ABa^	7048.93 ± 746.57^BCab^	9625.17 ± 400.80^Aa^	9682.15 ± 515.42^Aa^
	**15**	5511.32 ± 12.30^Ca^	6145.25 ± 242.49^BCb^	7129.53 ± 400.89^Bab^	8478.72 ± 102.03^Aab^	6894.06 ± 585.62^Bb^
**Springiness (mm)**	**0**	0.088 ± 0.01^ABCa^	0.065 ± 0.01^Ca^	0.119 ± 0.01^Aa^	0.111 ± 0.01^ABa^	0.080 ± 0.01^BCa^
	**3**	0.108 ± 0.03^Aa^	0.065 ± 0.01^Ba^	0.105 ± 0.01^ABa^	0.087 ± 0.01^ABab^	0.097 ± 0.01^ABa^
	**6**	0.102 ± 0.01^ABa^	0.074 ± 0.01^Ca^	0.090 ± 0.01^BCa^	0.086 ± 0.01^BCab^	0.115 ± 0.01^Aa^
	**9**	0.112 ± 0.01^Aa^	0.081 ± 0.01^Ba^	0.090 ± 0.01^ABa^	0.092 ± 0.01^ABab^	0.100 ± 0.01^ABa^
	**12**	0.089 ± 0.02^Aa^	0.079 ± 0.01^Aa^	0.090 ± 0.01^Aa^	0.089 ± 0.01^Aab^	0.096 ± 0.02^Aa^
	**15**	0.093 ± 0.02^Aa^	0.087 ± 0.01^Aa^	0.098 ± 0.01^Aa^	0.076 ± 0.01^Ab^	0.108 ± 0.02^Aa^
**Cohesiveness**	**0**	0.063 ± 0.01^Aba^	0.049 ± 0.01^Bc^	0.068 ± 0.01^Aa^	0.069 ± 0.01^Aa^	0.061 ± 0.01^ABa^
	**3**	0.069 ± 0.01^Aa^	0.066 ± 0.01^Aa^	0.060 ± 0.01^Aa^	0.055 ± 0.01^Aa^	0.068 ± 0.01^Aa^
	**6**	0.070 ± 0.01^Aa^	0.057 ± 0.01^Aabc^	0.057 ± 0.01^Aa^	0.063 ± 0.01^Aa^	0.073 ± 0.02^Aa^
	**9**	0.056 ± 0.01^Bab^	0.066 ± 0.01^Aba^	0.062 ± 0.01^ABa^	0.071 ± 0.01^Aa^	0.069 ± 0.01^ABa^
	**12**	0.031 ± 0.01^Bc^	0.062 ± 0.01^Aab^	0.054 ± 0.01^Aa^	0.058 ± 0.01^Aa^	0.064 ± 0.01^Aa^
	**15**	0.039 ± 0.01^Bbc^	0.052 ± 0.01^ABbc^	0.058 ± 0.01^Aa^	0.066 ± 0.01^Aa^	0.058 ± 0.01^Aa^
**Gumminess (N)**	**0**	408.24 ± 27.90^ABa^	277.60 ± 16.46^Bb^	308.50 ± 33.36^Ba^	477.05 ± 71.84^Aab^	462.76 ± 65.65^Aa^
	**3**	433.86 ± 86.39^Aa^	396.56 ± 24.03^ABab^	372.16 ± 28.25^ABa^	239.08 ± 46.75^Bb^	453.27 ± 55.89^Aa^
	**6**	411.99 ± 63.00^Aa^	422.68 ± 64.78^Aa^	403.77 ± 33.32^Aa^	510.86 ± 64.80^Aa^	456.25 ± 48.00^Aa^
	**9**	363.19 ± 92.18^Aa^	382.60 ± 14.87^Aab^	423.05 ± 53.62^Aa^	465.90 ± 116.28^Aab^	485.74 ± 71.42^Aa^
	**12**	284.03 ± 27.86^Aa^	482.83 ± 51.83^Aa^	356.90 ± 56.14^Aa^	493.96 ± 100.09^Aa^	502.36 ± 75.34^Aa^
	**15**	427.78 ± 27.17^Ba^	431.04 ± 63.83^Ba^	466.85 ± 63.66^ABa^	587.52 ± 38.57^Aa^	395.87 ± 34.41^Ba^
**Chewiness (N** × **mm)**	**0**	33.49 ± 6.87^ABbc^	18.72 ± 0.60^Bb^	34.36 ± 6.23^ABa^	49.91 ± 6.97^Aa^	40.27 ± 4.07^Aa^
	**3**	66.13 ± 14.23^Aa^	34.09 ± 1.46^Ba^	35.05 ± 7.39^Ba^	33.17 ± 3.44^Ba^	32.23 ± 4.68^Ba^
	**6**	46.32 ± 3.67^Aab^	25.50 ± 4.97^Bab^	23.18 ± 6.04^Ba^	22.49 ± 4.34^Ba^	34.91 ± 3.12^ABa^
	**9**	36.26 ± 16.30^Abc^	31.64 ± 1.88^Aa^	34.39 ± 6.82^Aa^	44.03 ± 8.67^Aa^	29.01 ± 2.81^Aa^
	**12**	10.54 ± 0.38^Bc^	29.74 ± 5.62^ABa^	20.90 ± 5.53^ABa^	27.54 ± 5.61^ABa^	34.72 ± 8.77^Aa^
	**15**	15.78 ± 1.99^Bc^	25.14 ± 2.35^Bab^	27.83 ± 3.03^Ba^	44.89 ± 4.79^Aa^	27.52 ± 4.02^Ba^
**Resilience**	**0**	0.034 ± 0.01^BCa^	0.027 ± 0.01^Cbc^	0.027 ± 0.01^Ca^	0.040 ± 0.01^ABa^	0.045 ± 0.01^Aa^
	**3**	0.039 ± 0.01^Aa^	0.035 ± 0.01^ABab^	0.026 ± 0.01^Ba^	0.028 ± 0.01^ABab^	0.036 ± 0.01^ABab^
	**6**	0.038 ± 0.01^Aa^	0.034 ± 0.01^Aabc^	0.033 ± 0.01^Aa^	0.033 ± 0.01^Aab^	0.037 ± 0.01^Aab^
	**9**	0.029 ± 0.01^Bab^	0.037 ± 0.01^ABa^	0.033 ± 0.01^Aba^	0.038 ± 0.01^Aa^	0.040 ± 0.01^Aab^
	**12**	0.020 ± 0.01^Cb^	0.034 ± 0.01^ABabc^	0.026 ± 0.01^BCa^	0.024 ± 0.01^Cb^	0.036 ± 0.01^Aab^
	**15**	0.022 ± 0.01^Bb^	0.026 ± 0.01^ABc^	0.033 ± 0.01^Aa^	0.033 ± 0.01^Aab^	0.030 ± 0.01^ABb^

**^A-C^:** The mean values with different letters in the same line and **^a– d^:** The mean values with different letters in the same column are significantly different (*P* < 0.05).

### Sensory attributes of the breast fillets

Marinating with rhubarb juice did not lead to any significant differences in the sensory properties of the chicken breast fillets compared to the non-marinated group (control), as shown in Table 5 (*P* > 0.05). As mentioned above, the rhubarb juice-based marinade significantly affected the texture and color characteristics of the raw breast fillets. However, the cooked fillets were rated as similar by the panel members in terms of texture and color characteristics (*P* > 0.05). In addition, the non-marinated fillets displayed indications of deterioration after the 9th d of storage, including a slimy surface and an abnormal odor. Therefore, no sensory evaluation was performed after the 9th d of storage. However, no signs of sensory deterioration were observed in the marinated samples during the entire storage period. The microbiological findings (Fig. 2, Fig. 3) and the volatiles profiles of the marinated breast fillets (Supplementary Table 1) also confirmed that psychrotrophic bacteria, TVC, LAB, alcohols, ketones, aldehydes and hydrocarbons increased significantly in the control group, while a slower growth of these microorganisms and an accumulation of these volatiles was observed in the marinated fillets. Considering these microbiological and volatile results, it is evident that they are consistent with the results of the sensory panel. Despite the presence of comparable psychrotrophs and LAB populations in the chicken breast fillets marinated with 50 % rhubarb juice for 24 h and in the control group after the sixth day of storage, no signs of sensory deterioration were observed in this group throughout the storage period. It can be assumed that the pronounced acidic composition and the aromatic compounds contained in the rhubarb juice have a significant influence on the sensory properties, which could be the cause of this phenomenon.

**Table 5:** Sensory attributes of the vacuum-packaged non-marinated and marinated with rhubarb juice chicken breast fillets during storage at 4 °C (mean ± SE).

Groups						
Storage (Days)		Control	50 % 6h	50 % 24h	100 % 6h	100 % 24h
**Color**	**0**	8.50 ± 0.34^Aa^	7.75 ± 0.75^Aa^	6.75 ± 0.75^Aa^	7.50 ± 0.65^Aa^	7.00 ± 0.77^Aa^
	**3**	8.67 ± 0.33^Aa^	7.33 ± 0.67^Aa^	7.00 ± 1.00^Aa^	7.50 ± 0.50^Aa^	7.00 ± 0.01^Aa^
	**6**	8.00 ± 0.01^Aa^	7.33 ± 0.33^Ba^	6.33 ± 0.33^Ca^	7.00 ± 0.01^Ba^	7.00 ± 0.01^Ba^
	**9**	8.00 ± 0.01^Aa^	6.60 ± 0.40^Ba^	6.80 ± 0.20^Ba^	7.20 ± 0.58^Aba^	6.40 ± 0.24^Ba^
	**12**	N.A.	7.50 ± 0.50^Aa^	7.50 ± 0.50^Aa^	7.50 ± 0.50^Aa^	6.50 ± 0.50^Aa^
	**15**	N.A.	7.00 ± 0.01^Aa^	7.00 ± 0.01^Aa^	7.00 ± 0.01^Aa^	6.50 ± 0.50^Aa^
**Odor**	**0**	8.20 ± 0.49^Aa^	7.60 ± 0.40^Aa^	7.60 ± 0.24^Aa^	7.66 ± 0.33^Aa^	7.50 ± 0.43^Aab^
	**3**	8.50 ± 0.50^Aa^	8.00 ± 0.01^Aa^	7.33 ± 0.33^Aa^	7.66 ± 0.33^Aa^	8.00 ± 0.58^Aa^
	**6**	8.25 ± 0.25^Aa^	7.75 ± 0.48^Aa^	7.67 ± 0.67^Aa^	7.75 ± 0.48^Aa^	7.67 ± 0.67^Aab^
	**9**	7.80 ± 0.20^Aa^	7.20 ± 0.37^Aba^	7.20 ± 0.37^Aba^	7.20 ± 0.37^ABab^	6.40 ± 0.24^Bab^
	**12**	N.A.	7.50 ± 0.50^Aa^	7.50 ± 0.50^Aa^	7.50 ± 0.50^Aab^	7.00 ± 0.01^Aab^
	**15**	N.A.	6.50 ± 0.50^Aa^	6.50 ± 0.50^Aa^	6.00 ± 1.00^Ab^	6.00 ± 1.00^Ab^
**Appearance**	**0**	8.33 ± 0.33^Aa^	7.75 ± 0.75^Aa^	6.60 ± 0.81^Aa^	7.50 ± 0.65^Aa^	7.61 ± 0.29^Aa^
	**3**	8.33 ± 0.33^Aa^	7.67 ± 0.33^Aba^	7.00 ± 0.01^Aba^	6.67 ± 0.33^Ba^	7.67 ± 0.33^Aba^
	**6**	8.00 ± 0.41^Aa^	7.33 ± 0.33^Aa^	6.67 ± 0.33^Aa^	7.00 ± 0.01^Aa^	7.00 ± 0.01^Aa^
	**9**	8.0 ± 0.32^Aa^	7.00 ± 0.45^Aa^	6.60 ± 0.25^Aa^	7.40 ± 0.40^Aa^	7.00 ± 0.01^Aa^
	**12**	N.A.	7.00 ± 0.01^Aa^	7.50 ± 0.50^Aa^	7.00 ± 0.01^Aa^	7.00 ± 0.01^Aa^
	**15**	N.A.	6.50 ± 0.50^Aa^	6.00 ± 1.00^Aa^	6.00 ± 1.00^Aa^	6.00 ± 1.00^Aa^
**Texture**	**0**	8.17 ± 0.40^Aa^	8.25 ± 0.48^Aa^	8.50 ± 0.50^Aa^	7.60 ± 0.68^Aa^	7.75 ± 0.48^Aa^
	**3**	7.67 ± 0.67^Aa^	6.67 ± 0.33^Aab^	7.00 ± 0.01^Aab^	6.33 ± 0.33^Aa^	7.33 ± 0.67^Aab^
	**6**	7.25 ± 0.63^Aa^	7.25 ± 0.48^Aab^	7.00 ± 0.41^Aab^	7.00 ± 0.41^Aa^	6.75 ± 0.48^Aab^
	**9**	7.00 ± 0.32^Aa^	6.75 ± 0.48^Aab^	6.20 ± 0.20^Ab^	6.80 ± 0.37^Aa^	6.00 ± 0.01^Aab^
	**12**	N.A.	7.00 ± 0.01^Aab^	7.50 ± 0.50^Aab^	7.00 ± 0.01^Aa^	5.50 ± 0.50^Bb^
	**15**	N.A.	6.50 ± 0.50^Ab^	6.50 ± 0.50^Aab^	6.00 ± 1.00^Aa^	5.50 ± 1.50^Ab^
**Flavor**	**0**	8.75 ± 0.25^Aa^	8.00 ± 0.41^Aa^	8.00 ± 0.58^Aa^	8.00 ± 0.58^Aa^	7.67 ± 0.33^Aab^
	**3**	8.00 ± 1.00^Aab^	7.00 ± 0.01^Aa^	7.00 ± 0.01^Aab^	6.50 ± 0.50^Aab^	8.00 ± 1.00^Aa^
	**6**	7.00 ± 0.41^Ab^	6.75 ± 0.48^Aa^	6.66 ± 0.33^Aab^	6.50 ± 0.29^Aab^	6.50 ± 0.29^Abc^
	**9**	7.80 ± 0.37^Aab^	7.00 ± 0.58^Aba^	6.40 ± 0.40^Bb^	7.00 ± 0.32^ABab^	6.25 ± 0.25^Bbc^
	**12**	N.A.	7.00 ± 1.00^Aa^	7.00 ± 0.01^Aab^	6.50 ± 0.50^Aab^	5.50 ± 0.50^Ac^
	**15**	N.A.	6.00 ± 1.00^Aa^	6.00 ± 0.01^Ab^	5.50 ± 0.50^Ab^	5.50 ± 0.50^Ac^
**Overall acceptability**	**0**	8.33 ± 0.33^Aa^	7.75 ± 0.25^Aa^	8.33 ± 0.33^Aa^	7.25 ± 0.48^Aa^	7.50 ± 0.29^Aa^
	**3**	7.33 ± 0.88^Aa^	6.66 ± 0.33^Aa^	7.00 ± 0.01^Aab^	6.33 ± 0.33^Aa^	7.00 ± 0.58^Aa^
	**6**	7.75 ± 0.25^Aa^	7.00 ± 0.58^Aa^	6.33 ± 0.67^Abc^	6.66 ± 0.33^Aa^	6.66 ± 0.33^Aa^
	**9**	7.60 ± 0.40^Aa^	6.80 ± 0.37^Aa^	6.60 ± 0.24^Abc^	6.80 ± 0.37^Aa^	6.50 ± 0.29^Aa^
	**12**	N.A.	7.50 ± 0.50^Aa^	7.00 ± 0.01^ABab^	6.50 ± 0.50^Aba^	6.00 ± 0.01^Ba^
	**15**	N.A.	6.50 ± 0.50^Aa^	5.50 ± 0.50^Ac^	6.00 ± 0.01^Aa^	6.00 ± 1.00^Aa^

**^A-C^:** The mean values with different letters in the same line, **^a-c^:** The mean values with different letters in the same column are significantly different (*P* < 0.05). N. A.: Not analysed.

## Conclusion

The results of the characterization analysis showed that rhubarb juice contains a large number of bioactive compounds involved in free radical scavenging and antimicrobial activities. In this context, it was found that the marinade based on rhubarb juice significantly delayed the oxidative changes and microbiological deterioration of chicken breast fillets without significant changes in sensory properties. In addition, the microbial safety of chicken breast fillets was improved by the inhibition of foodborne pathogenic bacteria, including *S*. Typhimurium, *L. monocytogenes* and *E. coli* O157:H7. In conclusion, the results of the present study show that the use of rhubarb juice as a marinating liquid can improve the chemical and microbiological quality and safety of breast fillets. Nevertheless, further studies are needed to elucidate the potential of rhubarb juice on the quality characteristics of different types of food.

## Declaration of competing interest

The authors declare that they have no known competing financial interests or personal relationships that could have appeared to influence the work reported in this paper.

## Appendix Supplementary materials

Image, application 1
