# Supplementary material for: The impact of rhubarb (Rheum Ribes L.) juice-based marinade on the quality characteristics and microbial safety of chicken breast fillets during refrigerated storage
Source: Poult Sci. 2024 Dec 21;104(2):104719. doi: 10.1016/j.psj.2024.104719 (PMC11742364; doi:10.1016/j.psj.2024.104719)
Supplement: Supplementary file 1 [file mmc1.docx]

| **Supplementary Table 1.** Changes in the volatiles in vacuum-packaged marinated with rhubarb juice and non-marinated chicken breast fillets during storage at 4 °C (mean peak area ± SE). | | | | | | | | | | | | | | | | | |
| --- | --- | --- | --- | --- | --- | --- | --- | --- | --- | --- | --- | --- | --- | --- | --- | --- | --- |
| **Groups** | | | | | | | | | | | | | | | | | |
|  | **Control** | | | | **50% 6h** | | | **50% 24h** | | | **100% 6h** | | | | **100% 24h** | | |
| **Storage (days)** | | | | | | | | | | | | | | | | | |
| ***Compounds*** | | **1** | **6** | **15** | **1** | **6** | **15** | **1** | **6** | **15** | **1** | **6** | | **15** | **1** | **6** | **15** |
| ***Alcohols*** | |  |  |  |  |  |  |  |  |  |  |  | |  |  |  |  |
| Ethanol | | 7.42±1.59 | 16.75±1.63 | 18.49±3.65 | 21.66±1.46 | 24.50±0.79 | 31.26±0.17 | 14.05±0.05 | 15.73±0.52 | 13.70±0.50 | 17.37±3.10 | 38.03±2.98 | | 46.92±4.33 | 26.72±2.06 | 20.87±2.01 | 42.78±0.16 |
| 1-Butanol | | 1.55±0.43 | 3.70±0.80 | 2.07±0.04 | 1.25±0.01 | 1.87±0.11 | 1.89±0.16 | 0.73±0.16 | 1.30±0.10 | 1.47±0.05 | 0.76±0.02 | 1.32±0.01 | | 1.81±0.23 | 1.60±0.20 | 1.26±0.10 | 2.09±0.10 |
| 1-Penten-3-ol | | 0.10±0.01 | 0.12±0.01 | 0.03±0.00 | 0.14±0.01 | 0.12±0.01 | 0.08±0.00 | 0.07±0.01 | 0.07±0.01 | 0.06±0.00 | 0.08±0.01 | 0.07±0.00 | | 0.08±0.01 | 0.11±0.01 | 0.15±0.02 | 0.12±0.01 |
| 3-Methyl-1-butanol | | 1.15±0.00 | 1.26±0.15 | 2.05±0.73 | 1.20±0.30 | 0.57±0.18 | 1.24±0.33 | 0.17±0.03 | 1.26±0.20 | 2.86±0.09 | 1.28±0.02 | 1.05±0.24 | | 3.55±0.58 | 1.26±0.03 | 1.33±0.27 | 1.99±0.13 |
| 2-Methyl-1-butanol | | 0.39±0.00 | 0.30±0.04 | 0.42±0.14 | 0.33±0.09 | 0.09±0.03 | 0.47±0.16 | 0.05±0.00 | 0.34±0.04 | 1.10±0.01 | 0.34±0.09 | 0.24±0.06 | | 1.28±0.15 | 0.28±0.04 | 0.30±0.13 | 0.46±0.04 |
| 1-Pentanol | | 0.44±0.00 | 0.35±0.04 | 0.39±0.16 | 0.47±0.10 | N.D. | N.D. | N.D. | 0.19±0.00 | 0.19±0.00 | 0.29±0.00 | 0.15±0.01 | | N.D. | N.D. | 0.34±0.03 | 0.34±0.00 |
| 3-Hexen-1-ol | | N.D. | N.D. | N.D. | 7.12±1.52 | 5.85±0.34 | 6.94±0.77 | 6.89±1.37 | 4.41±0.32 | 6.38±0.40 | 17.49±0.30 | 7.84±0.70 | | 8.95±1.42 | 12.91±2.33 | 8.56±0.57 | 15.70±1.52 |
| 1-Hexanol | | 0.24±0.14 | 0.20±0.04 | 0.59±0.20 | 0.76±0.13 | N.D. | N.D. | 0.35±0.07 | 0.40±0.02 | N.D. | 0.73±0.02 | N.D. | | N.D. | N.D. | N.D. | N.D. |
| 5-Ethyl-2-nonanol | | N.D. | 0.23±0.08 | 0.78±0.00 | 0.14±0.00 | N.D. | 0.10±0.02 | N.D. | 0.11±0.01 | 0.32±0.07 | 0.28±0.08 | 0.17±0.00 | | 0.19±0.04 | 0.18±0.00 | 0.18±0.02 | 0.51±0.09 |
| 1-Octen-3-ol | | 0.59±0.21 | 0.50±0.00 | 0.50±0.00 | 0.67±0.21 | 0.22±0.00 | 0.50±0.00 | N.D. | N.D. | N.D. | N.D. | N.D. | N.D. | | N.D. | 1.11±0.18 | N.D. |
| 2-Ethyl-hexanol | | 2.07±0.50 | 1.46±0.02 | 1.59±0.59 | 1.61±0.48 | 0.93±0.06 | 0.95±0.06 | 0.86±0.01 | 1.09±0.15 | 1.15±0.13 | 1.36±0.02 | 0.97±0.07 | 0.99±0.28 | | N.D. | N.D. | N.D. |
| Dimethyl-silanediol | | 0.78±0.00 | 1.18±0.24 | 1.88±0.19 | 1.23±0.09 | 0.86±0.04 | 0.73±0.04 | 1.21±0.43 | 0.80±0.04 | 0.64±0.09 | N.D. | 0.78±0.15 | 0.85±0.22 | | 0.85±0.13 | 0.68±0.11 | N.D. |
| ***Total (12)*** | | 14.74 | 26.05 | 28.79 | 36.58 | 35.01 | 44.15 | 24.39 | 25.68 | 27.88 | 39.97 | 50.62 | 64.62 | | 43.91 | 34.79 | 63.99 |
|  | |  |  |  |  |  |  |  |  |  |  |  |  | |  |  |  |
| ***Aldehydes*** | |  |  |  |  |  |  |  |  |  |  |  |  | |  |  |  |
| Acetaldehyde | | 0.69±0.00 | 1.73±0.00 | 1.33±0.15 | 1.54±0.01 | 1.14±0.15 | 1.31±0.13 | 1.16±0.29 | 1.33±0.00 | 1.07±0.00 | 0.63±0.04 | 1.00±0.33 | 2.11±0.42 | | 1.51±0.59 | 2.24±0.00 | 2.88±0.07 |
| 3-Methyl-butanal | | 0.02±0.00 | 0.03±0.00 | 0.28±0.04 | 0.15±0.08 | 0.40±0.09 | 0.72±0.13 | 0.04±0.00 | 0.35±0.09 | 0.67±0.01 | 0.05±0.01 | 0.21±0.03 | 1.01±0.13 | | 0.26±0.11 | 0.81±0.12 | 1.74±0.01 |
| Paraldehyde | | N.D. | 0.30±0.08 | 0.54±0.16 | 0.09±0.00 | 0.05±0.01 | 0.08±0.00 | N.D. | 0.15±0.01 | 0.26±0.07 | 0.24±0.00 | 0.19±0.00 | 0.27±0.01 | | 0.17±0.00 | 0.17±0.02 | 0.97±0.16 |
| Hexanal | | 1.17±0.28 | 1.46±0.19 | 2.06±0.49 | 2.74±0.23 | 1.62±0.04 | 4.51±0.00 | 0.38±0.10 | 1.11±0.33 | 2.32±0.19 | 1.90±0.08 | 1.47±0.18 | 2.79±0.30 | | 2.53±0.57 | 18.93±1.33 | 11.40±1.37 |
| Heptanal | | N.D. | N.D. | N.D. | 0.15±0.00 | N.D. | 0.12±0.00 | 0.03±0.00 | N.D. | 0.16±0.00 | 0.18±0.11 | N.D. | N.D. | | 0.17±0.02 | 0.44±0.10 | 0.56±0.08 |
| Benzaldehyde | | N.D. | N.D. | N.D. | N.D. | N.D. | N.D. | N.D. | N.D. | 0.14±0.02 | 0.08±0.00 | N.D. | 0.13±0.06 | | 0.07±0.00 | 0.19±0.02 | 0.35±0.13 |
| Octanal | | 0.12±0.04 | N.D. | N.D. | 0.11±0.00 | N.D. | 0.59±0.00 | N.D. | N.D. | N.D. | 0.15±0.04 | N.D. | N.D. | | N.D. | 0.23±0.06 | 0.18±0.06 |
| Nonanal | | 0.46±0.09 | 0.25±0.03 | 0.44±0.21 | 0.38±0.03 | 0.11±0.00 | 0.24±0.01 | 0.14±0.00 | 0.18±0.06 | 0.39±0.01 | 0.55±0.12 | 0.13±0.01 | 0.29±0.03 | | 0.31±0.11 | 0.47±0.13 | 0.64±0.10 |
| ***Total (8)*** | | 2.46 | 3.77 | 4.65 | 5.17 | 3.33 | 7.56 | 1.76 | 3.12 | 5.01 | 3.79 | 3.00 | 6.59 | | 5.02 | 23.49 | 18.73 |
|  | |  |  |  |  |  |  |  |  |  |  |  |  | |  |  |  |
| ***Esters*** | |  |  |  |  |  |  |  |  |  |  |  |  | |  |  |  |
| Methyl acetate | | 0.54±0.18 | 1.31±0.15 | 4.71±0.86 | 0.98±0.21 | N.D. | 4.16±0.46 | 0.45±0.28 | 2.24±0.23 | 9.38±0.26 | 0.61±0.14 | 0.79±0.16 | 11.30±2.16 | | 4.12±1.05 | 2.12±0.33 | 6.39±1.60 |
| Ethyl acetate | | 8.12±0.00 | 9.76±0.50 | 16.97±2.86 | 17.36±0.54 | 6.57±1.05 | 28.84±2.28 | 2.35±0.37 | 9.39±2.19 | 20.69±0.30 | 11.56±1.90 | 25.34±5.92 | 51.01±3.18 | | 21.81±0.00 | 15.22±0.03 | 53.83±4.55 |
| Methyl butanoate | | 0.19±0.00 | 0.43±0.06 | 0.58±0.08 | 0.36±0.09 | 0.20±0.00 | 0.55±0.00 | 0.14±0.00 | 0.43±0.08 | 1.00±0.19 | 0.70±0.26 | 0.32±0.04 | 1.01±0.22 | | 0.66±0.25 | 0.42±0.00 | 0.80±0.14 |
| Hexyl formate | | N.D. | N.D. | N.D. | N.D. | 0.52±0.04 | 0.72±0.04 | N.D. | N.D. | 0.86±0.16 | N.D. | 0.69±0.17 | 0.84±0.15 | | 0.65±0.16 | 1.06±0.01 | 0.98±0.33 |
| 1-Methyl-1-propenyl acetate | | N.D. | 0.45±0.13 | 1.09±0.44 | N.D. | 0.06±0.00 | 0.20±0.03 | N.D. | 0.28±0.00 | 0.45±0.10 | 0.27±0.00 | 0.15±0.00 | 0.33±0.08 | | 0.25±0.00 | 0.18±0.01 | 0.83±0.25 |
| Methyl hexanoate | | 0.15±0.00 | 0.44±0.11 | 1.38±0.00 | 0.16±0.08 | 0.14±0.00 | 0.19±0.02 | N.D. | 0.14±0.04 | 0.41±0.10 | 0.24±0.13 | 0.19±0.00 | 0.21±0.07 | | 0.22±0.04 | 0.31±0.03 | 0.66±0.10 |
| Ethyl hexanoate | | 0.65±0.00 | 0.95±0.27 | 1.37±0.11 | 0.43±0.13 | 0.35±0.01 | 0.66±0.00 | 0.17±0.00 | 0.60±0.07 | 1.00±0.17 | 0.72±0.26 | 0.64±0.25 | 0.65±0.09 | | 0.46±0.14 | 0.96±0.09 | 1.40±0.40 |
| Methyl octanoate | | 0.26±0.00 | 0.12±0.01 | 0.30±0.00 | 0.07±0.00 | N.D. | N.D. | N.D. | 0.10±0.00 | 0.11±0.01 | 0.14±0.01 | N.D. | 0.11±0.00 | | 0.13±0.00 | N.D. | 0.21±0.00 |
| ***Total (8)*** | | 9.91 | 13.46 | 26.41 | 19.36 | 7.85 | 35.32 | 3.10 | 13.17 | 33.92 | 14.24 | 28.12 | 65.45 | | 28.29 | 20.26 | 65.09 |
|  | |  |  |  |  |  |  |  |  |  |  |  |  | |  |  |  |
| ***Hydrocarbons*** | |  |  |  |  |  |  |  |  |  |  |  |  | |  |  |  |
| 2-Methyl-3-(methylthio)-butane | | 0.97±0.42 | 0.85±0.11 | 0.33±0.00 | 1.81±0.07 | 1.70±0.15 | 1.43±0.30 | 1.74±0.26 | 1.27±0.45 | 1.22±0.31 | 1.74±0.01 | 1.12±0.23 | 1.37±0.52 | | 1.09±0.00 | 1.06±0.16 | 1.80±0.22 |
| 3-Methyl-pentane | | 0.62±0.25 | 0.17±0.04 | 0.10±0.01 | 0.37±0.14 | 0.48±0.01 | 0.35±0.03 | 0.71±0.27 | 0.21±0.01 | 0.23±0.08 | 0.35±0.10 | 0.22±0.08 | 0.27±0.12 | | 0.85±0.21 | 0.25±0.03 | 0.24±0.08 |
| Heptane | | 0.13±0.07 | 0.12±0.02 | 0.16±0.06 | 0.26±0.06 | 0.05±0.05 | 0.18±0.02 | N.D. | 0.11±0.00 | 0.14±0.01 | 0.22±0.03 | 0.09±0.00 | 0.18±0.05 | | 0.45±0.13 | 0.59±0.08 | 0.87±0.01 |
| Chloro benzene | | N.D. | 0.16±0.04 | 0.27±0.00 | 0.14±0.00 | N.D. | 0.11±0.04 | N.D. | 0.08±0.00 | 0.19±0.02 | 0.14±0.04 | 0.20±0.00 | 0.21±0.03 | | 0.15±0.00 | 0.15±0.02 | 0.37±0.13 |
| Ethyl benzene | | 0.20±0.05 | 0.19±0.04 | 0.24±0.16 | 0.26±0.08 | 0.74±0.05 | 0.63±0.04 | 0.51±0.00 | 0.55±0.08 | 0.73±0.08 | 0.09±0.06 | 1.30±0.30 | 0.91±0.11 | | 0.65±0.15 | 0.83±0.01 | 0.81±0.22 |
| o-Xylene | | 0.82±0.23 | 0.29±0.02 | 0.35±0.14 | 0.52±0.20 | 1.14±0.04 | 0.62±0.08 | 0.19±0.10 | 0.37±0.07 | 0.71±0.07 | 0.31±0.06 | 0.35±0.00 | 0.85±0.14 | | 0.73±0.13 | 0.75±0.01 | 0.75±0.18 |
| Styrene | | 0.51±0.00 | 1.25±0.40 | 1.78±0.22 | 0.76±0.57 | 0.53±0.03 | 0.97±0.06 | 0.16±0.00 | 0.77±0.08 | 1.84±0.40 | 1.15±0.30 | 0.93±0.16 | 1.40±0.17 | | 0.81±0.06 | 1.23±0.17 | 2.51±0.55 |
| Dodecane | | 0.07±0.00 | 0.11±0.04 | 0.29±0.00 | 0.04±0.00 | N.D. | 0.05±0.01 | N.D. | 0.05±0.01 | 0.11±0.03 | 0.05±0.03 | 0.11±0.00 | 0.05±0.00 | | 0.07±0.00 | 0.07±0.01 | 0.13±0.04 |
| ***Total (8)*** | | 3.32 | 3.15 | 3.52 | 4.15 | 4.64 | 4.33 | 3.30 | 3.42 | 5.16 | 4.05 | 4.32 | 5.24 | | 4.79 | 4.92 | 7.5 |
|  | |  |  |  |  |  |  |  |  |  |  |  |  | |  |  |  |
| ***Ketones*** | |  |  |  |  |  |  |  |  |  |  |  |  | |  |  |  |
| 2,3-Butanedione (Diacetyl) | | 0.04±0.00 | 0.03±0.00 | 0.20±0.05 | 0.04±0.00 | 0.07±0.01 | 0.22±0.08 | N.D. | 0.13±0.01 | 0.49±0.07 | 0.06±0.02 | 0.02±0.00 | 0.28±0.12 | | 0.14±0.01 | 0.09±0.04 | 0.36±0.18 |
| 2-Pentanone | | 0.14±0.08 | 0.30±0.04 | 0.24±0.01 | 0.29±0.05 | 0.33±0.01 | 0.40±0.05 | 0.23±0.00 | 0.30±0.07 | 0.34±0.01 | 0.21±0.01 | 0.26±0.04 | 0.39±0.02 | | 0.57±0.13 | 0.35±0.03 | 0.36±0.01 |
| 3-Hydroxy- 2-butanone (Acetoin) | | N.D. | N.D. | 0.17±0.00 | N.D. | N.D. | 1.37±0.37 | N.D. | 1.06±0.00 | 0.60±0.00 | N.D. | N.D. | 0.95±0.00 | | N.D. | 0.74±0.00 | 4.37±1.03 |
| 5-Methyl-2-hexanone | | 0.68±0.00 | 0.52±0.14 | 0.63±0.14 | N.D. | N.D. | N.D. | N.D. | N.D. | N.D. | N.D. | N.D. | N.D. | | N.D. | N.D. | N.D. |
| 3-Heptanone | | 0.07±0.00 | 0.19±0.04 | 0.35±0.28 | 0.13±0.00 | 0.05±0.00 | 0.13±0.00 | N.D. | 0.12±0.00 | 0.26±0.06 | 0.14±0.04 | 0.17±0.00 | 0.20±0.06 | | 0.15±0.00 | 0.15±0.01 | 0.39±0.08 |
| 2-Heptanone | | 0.37±0.00 | 0.28±0.04 | 0.67±0.00 | 0.26±0.10 | 0.17±0.03 | 0.32±0.02 | 0.07±0.00 | 0.31±0.08 | 0.47±0.08 | 0.32±0.02 | 0.25±0.04 | 0.42±0.08 | | 0.24±0.07 | 0.41±0.04 | 0.60±0.18 |
| 3-Methyl-hexan-2-one | | N.D. | 0.37±0.10 | 0.87±0.19 | N.D. | 0.07±0.00 | 0.17±0.02 | N.D. | 0.22±0.00 | 0.34±0.07 | 0.22±0.00 | 0.14±0.00 | 0.31±0.08 | | 0.21±0.00 | 0.15±0.01 | 0.62±0.18 |
| 2-Methyl-heptan-3-one | | N.D. | 0.72±0.19 | 2.48±0.00 | N.D. | N.D. | 0.50±0.00 | N.D. | N.D. | 0.90±0.23 | 0.65±0.00 | 0.59±0.00 | 0.74±0.00 | | N.D. | N.D. | 1.35±0.25 |
| 6-Methyl-5-hepten-2-one | | 0.95±0.25 | 0.81±0.18 | 1.27±0.00 | 0.78±0.00 | 0.40±0.01 | 0.62±0.00 | 0.23±0.00 | 0.69±0.11 | 0.94±0.12 | 1.47±0.03 | 0.84±0.06 | 0.93±0.00 | | 2.65±0.71 | 1.65±0.18 | 1.04±0.21 |
| ***Total (9)*** | | 2.26 | 3.23 | 6.87 | 1.49 | 1.07 | 3.73 | 0.54 | 2.82 | 4.34 | 3.07 | 2.26 | 4.21 | | 3.96 | 3.54 | 9.09 |
|  | |  |  |  |  |  |  |  |  |  |  |  |  | |  |  |  |
| ***Terpens*** | |  |  |  |  |  |  |  |  |  |  |  |  | |  |  |  |
| Oxime-methoxy-phenyl | | 0.51±0.00 | 1.32±0.00 | 1.26±0.15 | 0.79±0.34 | N.D. | 0.63±0.00 | 0.28±0.00 | 0.23±0.00 | 0.86±0.00 | 0.09±0.00 | 0.83±0.00 | 1.63±0.00 | | N.D. | N.D. | 1.30±0.20 |
| 𝛼-Phellandrene | | 0.09±0.00 | 0.10±0.02 | 0.33±0.00 | N.D. | 0.05±0.00 | 0.17±0.00 | N.D. | N.D. | 0.13±0.00 | N.D. | N.D. | 0.23±0.00 | | 0.15±0.05 | 0.14±0.02 | 0.24±0.11 |
| 𝛼-Pinene | | 1.26±0.00 | 1.06±0.21 | 1.48±0.31 | 0.93±0.22 | 1.06±0.08 | 1.21±0.24 | 0.61±0.00 | 1.36±0.18 | 1.23±0.15 | 0.94±0.01 | 1.07±0.08 | 1.47±0.08 | | 2.44±0.58 | 1.92±0.08 | 1.72±0.16 |
| Sabinene | | N.D. | 0.69±0.00 | 1.03±0.00 | N.D. | 0.26±0.00 | 0.48±0.00 | 0.10±0.00 | 0.25±0.05 | 0.45±0.04 | 0.51±0.01 | 0.23±0.02 | 0.37±0.09 | | 0.25±0.01 | N.D. | 0.65±0.13 |
| 𝛽-Pinene | | 0.71±0.00 | 0.61±0.12 | 0.65±0.14 | 0.63±0.06 | 0.53±0.03 | 0.86±0.00 | 0.23±0.00 | 0.50±0.08 | 0.76±0.12 | 0.54±0.01 | 0.56±0.00 | 0.70±0.08 | | 0.84±0.21 | 2.34±0.35 | 1.31±0.04 |
| 𝛽-Myrcene | | N.D. | 2.14±0.38 | 2.32±0.54 | 1.53±0.00 | 1.25±0.08 | 1.59±0.09 | 0.74±0.00 | 1.53±0.17 | 2.15±0.38 | 2.11±0.00 | 1.75±0.47 | 2.00±0.37 | | 1.72±0.07 | 2.46±0.07 | 3.01±0.57 |
| 𝛽-Ocimene | | 0.12±0.00 | 0.12±0.02 | 0.11±0.08 | 0.09±0.01 | 0.11±0.01 | 0.12±0.00 | 0.06±0.00 | 0.11±0.01 | 0.14±0.02 | 0.10±0.01 | 0.11±0.02 | 0.13±0.01 | | 0.16±0.05 | 0.16±0.01 | 0.19±0.04 |
| p-Cymene | | N.D. | N.D. | N.D. | N.D. | N.D. | N.D. | N.D. | N.D. | N.D. | N.D. | N.D. | N.D. | | 0.89±0.28 | 1.06±0.11 | 1.35±0.62 |
| dl-Limonene | | 43.67±0.00 | 82.30±19.43 | 98.75±24.64 | 34.80±5.21 | 50.12±4.24 | 67.31±1.63 | 27.15±0.00 | 58.61±6.41 | 91.76±7.77 | 55.78±8.69 | 70.49±7.67 | 71.55±7.86 | | 59.69±4.42 | 85.54±3.69 | 121.19±23.97 |
| 1,8-Cineole | | 2.40±0.60 | 4.23±1.41 | 5.65±1.38 | 1.90±0.05 | 1.68±0.06 | 3.40±0.32 | 1.22±0.00 | 4.00±1.07 | 5.13±1.13 | 3.16±0.07 | 2.90±0.50 | 4.14±1.07 | | 2.59±0.77 | 3.71±0.65 | 7.33±1.05 |
| 𝛾-Terpinene | | 0.25±0.00 | 0.19±0.06 | 0.19±0.15 | 0.12±0.03 | 0.11±0.00 | 0.16±0.01 | 0.04±0.00 | 0.17±0.03 | 0.21±0.05 | 0.20±0.02 | 0.20±0.06 | 0.17±0.02 | | 0.14±0.04 | 0.23±0.01 | 0.28±0.07 |
| Linalool | | 1.39±0.13 | 1.47±0.41 | 2.19±0.86 | 0.42±0.04 | 0.20±0.01 | 0.65±0.16 | 0.07±0.01 | 0.83±0.23 | 1.24±0.05 | 1.39±0.42 | 0.53±0.14 | 0.93±0.08 | | 0.70±0.13 | 0.97±0.03 | 1.17±0.35 |
| ***Total (12)*** | | 50.40 | 94.23 | 113.95 | 41.20 | 55.39 | 76.57 | 30.49 | 67.59 | 104.06 | 64.83 | 78.67 | 83.32 | | 69.58 | 98.53 | 139.73 |
|  | |  |  |  |  |  |  |  |  |  |  |  |  | |  |  |  |
| ***Miscellaneous compounds*** | |  |  |  |  |  |  |  |  |  |  |  |  | |  |  |  |
| Carbon disulfide | | 0.40±0.18 | 0.13±0.07 | 0.91±0.65 | 0.18±0.06 | 0.44±0.04 | 0.95±0.11 | 0.35±0.00 | 0.74±0.25 | 1.67±0.00 | 0.17±0.01 | 0.70±0.07 | N.D. | | 0.05±0.00 | 0.40±0.07 | 1.73±0.00 |
| Diisopropyl ether | | 5.71±0.37 | 23.05±0.71 | 19.67±0.36 | 11.19±1.87 | 16.17±0.18 | 16.62±1.05 | 15.35±0.00 | 20.41±1.04 | 22.25±2.29 | 10.97±1.70 | 13.00±2.92 | 20.99±2.21 | | 23.87±0.00 | 20.71±0.68 | 30.02±4.14 |
| Tetrahydro-furan | | 1.35±0.49 | 3.31±0.81 | 2.62±0.33 | 3.59±1.01 | 3.36±0.25 | 5.31±0.93 | 4.36±0.00 | 2.88±0.42 | 5.19±1.36 | 1.71±0.26 | 3.71±0.36 | 4.61±0.45 | | 4.65±0.00 | 3.74±0.72 | 8.50±1.51 |
| 2,4-Dimethyl-acetophenone | | 0.16±0.00 | 0.07±0.00 | 0.22±0.00 | 0.15±0.08 | 0.09±0.00 | N.D. | N.D. | 0.22±0.00 | 0.14±0.00 | 0.12±0.03 | 0.10±0.00 | 0.13±0.00 | | 0.14±0.05 | 0.12±0.01 | 0.36±0.13 |
| ***Total (4)*** | | 7.62 | 26.56 | 23.42 | 15.12 | 20.06 | 22.89 | 20.06 | 24.24 | 29.24 | 12.96 | 17.52 | 25.74 | | 28.71 | 24.97 | 40.61 |
| N.D.: Not determined | | | | | | | | | | | | | | | | | |
